# SLO potassium channels antagonize premature decision making in *C. elegans*

**DOI:** 10.1038/s42003-018-0124-5

**Published:** 2018-08-24

**Authors:** Ichiro Aoki, Michihiro Tateyama, Takushi Shimomura, Kunio Ihara, Yoshihiro Kubo, Shunji Nakano, Ikue Mori

**Affiliations:** 1 Neuroscience Institute of the Graduate School of Science, Nagoya University, Nagoya, 464-8602 Japan; 2 Division of Biophysics and Neurobiology, Department of Molecular and Cellular Physiology, National Institute for Physiological Sciences, Okazaki, 444-8585 Japan; 3 Center for Gene Research, Nagoya University, Nagoya, 464-8602 Japan; 4 Group of Molecular Neurobiology, Graduate School of Science, Nnagoya University, Nagoya, 464-8602 Japan

## Abstract

Animals must modify their behavior with appropriate timing to respond to environmental changes. Yet, the molecular and neural mechanisms regulating the timing of behavioral transition remain largely unknown. By performing forward genetics to reveal mechanisms that underlie the plasticity of thermotaxis behavior in *C. elegans*, we demonstrated that SLO potassium channels and a cyclic nucleotide-gated channel, CNG-3, determine the timing of transition of temperature preference after a shift in cultivation temperature. We further revealed that SLO and CNG-3 channels act in thermosensory neurons and decelerate alteration in the responsiveness of these neurons, which occurs prior to the preference transition after a temperature shift. Our results suggest that regulation of sensory adaptation is a major determinant of latency before animals make decisions to change their behavior.

## Abstract (web summary)

Ichiro Aoki et al. demonstrate that a K+ channel and a cyclic nucleotide-gated channel regulate the adaptation of thermosensory neurons to temperature fluctuations in *C. elegans*. This study deepens our understanding of how long it takes for animals to accommodate environmental changes behaviorally.

## Introduction

One of the central issues in decision-making research is what determines the timing of behavior transition, during which animals learn from a new experience and abandon old knowledge^1,2^. Appropriate latency before behavior transition is necessary for animals to discriminate long-lasting environmental changes from temporary changes. For instance, animals keep the state of hibernation irrespective of daily change but emerge from it in response to seasonal change^3^. Nevertheless, if the latency is too long, animals lose opportunities to obtain rewards or fail to avoid dangers. Although regulation of the timing of behavior transition in response to environmental changes is critical for animals, the underlying molecular and neural principles of this process remain largely unknown.

**Fig. 1:**
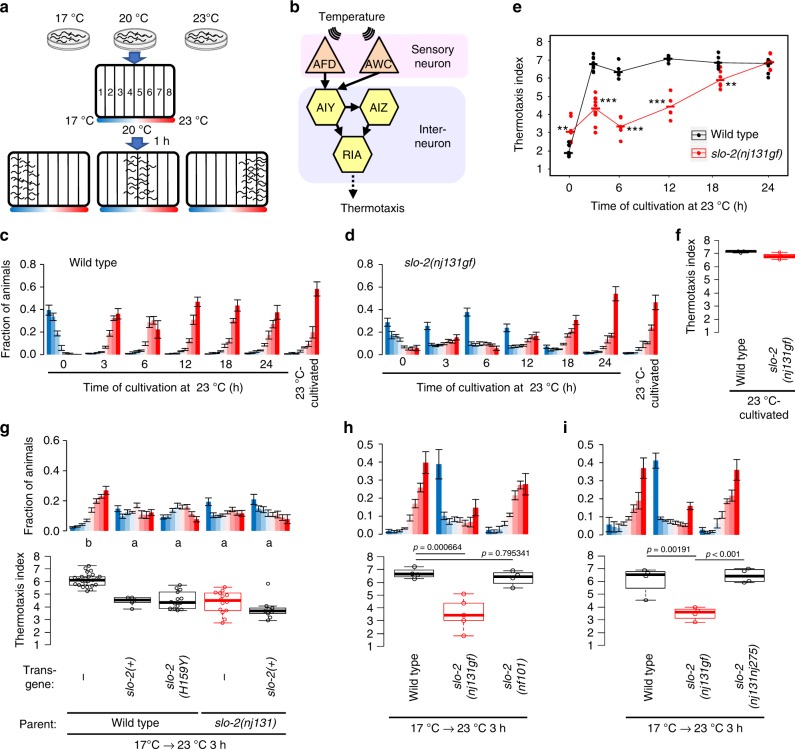
Gain of SLO-2 function decelerates transition of preference in thermotaxis behavior. **a** A scheme for a thermotaxis assay is shown. *C. elegans* cultivated at a certain temperature is placed at the center of a linear thermal gradient without food and is allowed to freely migrate for 1 h. **b** A neural circuit regulating thermotaxis is shown. **c**, **d** Wild-type (**c**) and *slo-2(nj131gf)* (**d**) animals were cultivated at 17 °C for 5 days and then at 23 °C for the time indicated or constantly at 23 °C for 3 days. The animals were then placed on a thermal gradient. The number of animals in each section of the thermal gradient was determined, and the proportion of animals in each region was plotted on a histogram. *n* = 6, 10, 6, 6, 6, 6, 3 for each time point (**c**, **d**). The error bars represent the standard error of mean (SEM). **e** The thermotaxis indices at each time point (**c**, **d**) were plotted against time after the cultivation temperature was changed to 23 °C. Horizontal bars indicate medians. **p < 0.01, ***p < 0.001 (Welch two-sample *t* test). **f** The thermotaxis indices are shown for animals cultivated constantly at 23 °C in **c**, **d**. **g** Genomic PCR fragments covering *slo-2* gene locus that were derived from either wild-type or *nj131* mutant animals were injected into either wild-type or *nj131* animals. Animals were cultivated at 17 °C for 5 days and at 23 °C for 3 h and then subjected to thermotaxis assay, as described above. Animals with extra chromosomal arrays were scored to evaluate thermotaxis. The fractions of animals were plotted on histograms (upper), and the thermotaxis indices were shown on boxplots (lower). *n* = 24, 5, 14, 14, 9 for each strain. The indices of strains marked with distinct alphabets differ significantly (*p* < 0.001) according to Tukey–Kramer test. **h**, **i** Wild-type and indicated *slo-2* mutant animals were cultivated at 17 °C for 5 days and at 23 °C for 3 h and then subjected to thermotaxis assay. *n* = 4 or 5. *p* Values are indicated (Dunnett test against wild-type (**h**) or Tukey–Kramer test (**i**)). See also Supplementary Fig. 1

To understand how the timing of behavior transition is determined, we examined the plasticity of the thermotaxis behavior of *Caenorhabditis elegans* (i.e., association of cultivation temperature with the existence of food and migration toward that temperature on a thermal gradient with no food)^4,5^ (Fig. 1a). The thermotaxis of *C. elegans* is essentially regulated by a simple neural circuit^4,6^ (Fig. 1b). AFD is a major thermosensory neuron in this circuit that increases the intracellular concentration of Ca^2+^ ([Ca^2+^]_i_) in response to a rise in temperature above the past cultivation temperature^7–11^. Since this cultivation temperature dependency in the AFD dynamic range is well conserved, even when AFD cells are cultured in isolation from the neural network in vitro, AFD cell-autonomously encodes information regarding cultivation temperature^8^. Genetic analyses have revealed the molecular components involved in temperature sensation in AFD. Three receptor-type guanylyl cyclases, GCY-8, GCY-18, and GCY-23, are specifically localized to the sensory ending of AFD and are thought to act as thermosensors^12,13^. These guanylyl cyclases synthesize cGMP, which activates cyclic nucleotide-gated (CNG) channels composed of TAX-2 and TAX-4^14,15^, leading to an influx of Ca^2+^ into AFD.

Thermotaxis behavior is plastic; when cultivation temperature shifts, animals change their temperature preference to the new cultivation temperature over the course of a few hours^5^ (Fig. 1c, e). Further, AFD neurons that are essential for thermotaxis acclimate to a new temperature by changing the dynamic range of responsive temperature^8,16^. However, the molecular mechanisms underlying the transition of the temperature preference and the AFD dynamic range are unknown.

In this study, we performed a forward genetic screen for mutants that were slow to change their temperature preference in thermotaxis and demonstrated that a gain-of-function (gf) mutation in the SLO-2 K^+^ channel decelerated the preference transition. The *slo-1* gene encodes the other member of the SLO family of K^+^ channel in *C. elegans*. Interestingly, the preference transition was accelerated in animals with *slo-2* and *slo-1* loss-of-function (lf) mutations. Calcium imaging of AFD revealed that SLO K^+^ channels slowed down AFD adaptation after an upshift in cultivation temperature. Moreover, a forward genetic screen for the suppressors of *slo-2gf* animals revealed that CNG-3, a subunit of CNG channels, cooperated with SLO-2 to decelerate both AFD adaptation and temperature preference transition.

It was recently reported that gf mutations in a human SLO-2 homolog, Slack/KCNT1, cause early-onset epilepsy^17,18^. We found that some epilepsy-related mutations potentiated *C. elegans* SLO-2 in decelerating temperature preference transition. These results imply that the early-onset epilepsy and the latency regulation in *C. elegans* thermotaxis might have similar molecular and physiological mechanisms.

## Results

### Isolation of *slo-2 gf* mutant that is slow to change behavior

To reveal the molecular mechanisms that determine the timing of behavior transition, we performed a forward genetic screen for mutants that were slow to change their temperature preference after a shift in cultivation temperature (Supplementary Fig. 1a). One of the mutants we isolated was *nj131*. When cultivated first at 17 °C and then re-cultivated at 23 °C for 3 h, wild-type animals migrated to a warm region, suggesting that 3 h of cultivation at a new temperature is sufficient to change their temperature preference. By contrast, a majority of *nj131* animals that were cultivated first at 17 °C and then at 23 °C for 3 h still migrated to a cold region, showing the old temperature preference (Fig. 1d). Nevertheless, *nj131* mutants eventually migrated to a warm region like the wild-type animals approximately 24 h after being transferred from 17 °C to 23 °C (Fig. 1d, e). When *nj131* mutants were cultivated constantly at 23 °C, they migrated to the warm region like the wild-type animals (Fig. 1d, f), suggesting that *nj131* mutants were not simply cryophilic but slow to change their behavior. Hereafter, we designate this abnormality of *nj131* mutants as a “slow-learning” (Slw) phenotype. When *nj131* mutants were first cultivated at 23 °C and then at 17 °C for 3 h, they migrated to a cold region, as did the wild-type animals (Supplementary Fig. 1c–e). This result suggests that *nj131* mutants exhibit the Slw phenotype specifically in response to temperature upshift.

Single-nucleotide polymorphism (SNP) mapping and whole-genome sequencing revealed that *nj131* was a missense mutation in the *slo-2* gene, which alters histidine residue 159 of the gene product to tyrosine (H159Y) (Supplementary Fig. 1b). We then examined whether *slo-2* expression can rescue the Slw phenotype in *nj131* mutants. Whereas injection of a PCR fragment containing *slo-2* genomic DNA into *nj131* animals did not rescue the Slw phenotype, introduction of mutant or wild-type forms of SLO-2 into wild-type animals phenocopied *nj131* mutants (Fig. 1g), suggesting that *nj131* is a gf allele of the *slo-2* gene. When cultivated constantly at 23 °C, both animals expressing the mutant form of SLO-2 and the wild-type animals migrated to a warm region (Supplementary Fig. 1f), suggesting that SLO-2 overexpression did not make animals simply cryophilic but phenocopied the *nj131* mutants. Consistent with that *nj131* is a gf allele of *slo-2*, *slo-2(nf101)* deletion mutants^19^ (Supplementary Fig. 1b) did not exhibit the Slw phenotype and migrated to a warm region like the wild-type animals when cultivated first at 17 °C then at 23 °C for 3 h (Fig. 1h). If the Slw phenotype of *nj131* mutants is truly caused by gf mutation of *slo-2*, knocking out the *slo-2* gene in *nj131* mutant animals should cancel the Slw phenotype. We indeed found that knocking out the *slo-2* gene using CRISPR/Cas9 clearly eliminated the expression of the Slw phenotype in *nj131* mutants (Fig. 1i), indicating that the Slw phenotype of *nj131* mutants was caused by gf mutation of the *slo-2* gene.

**Fig. 2:**
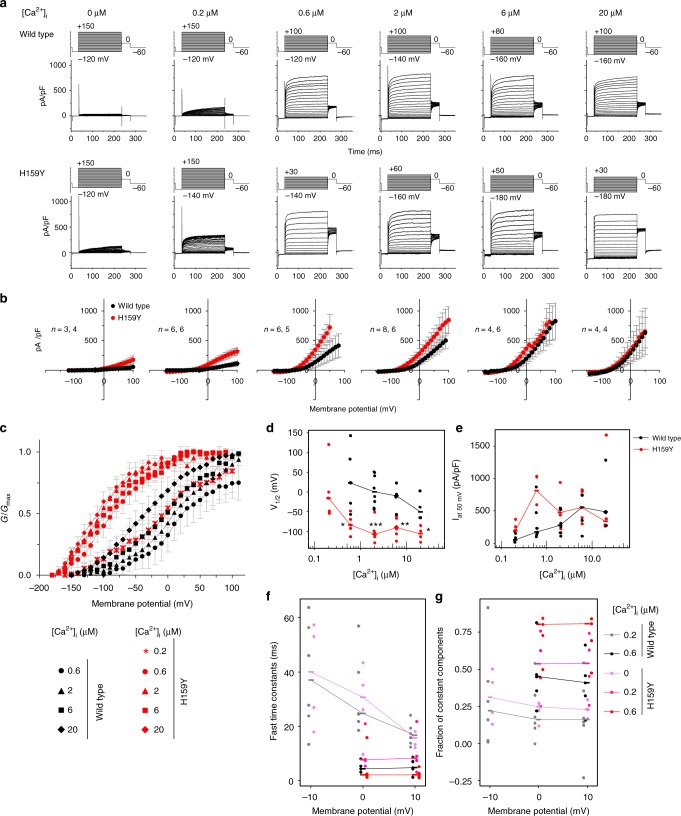
The channel currents of mutant SLO-2 (H159Y) are larger than those of wild type. **a** Representative whole-cell current recordings of HEK293T cells expressing either wild-type (*n* = 3, 6, 6, 8, 4, 4) or H159Y mutant (*n* = 4, 5, 5, 6, 6, 4) SLO-2 channels by patch clamp method are shown. Cells were held at −60 mV and depolarizing step pulses (200 ms) were applied as indicated above the current traces. Intracellular Ca^2+^ concentration ([Ca^2+^]_i_) is indicated above the current traces. **b** Current densities were measured at the end of step pulses and averaged for each membrane potential and [Ca^2+^]_i_ and are plotted against membrane potential. Currents were recorded in distinct cells, and the numbers of replications for wild-type and H159Y channels are indicated. The error bars represent the SEM. **c** Conductance (*G*) was measured as the tail current amplitude at 0 mV after various step-pulse stimulations and was normalized to the recorded maximal amplitude at the most depolarized potential (*G*_max_). Normalized conductance (*G*/*G*_max_) is plotted against membrane potential. The meaning of the symbols is indicated in the figure. The error bars represent the SEM. **d** The voltage at half-maximal activation(*V*_1/2_) of wild-type and H159Y mutant channels calculated from **c** is plotted against [Ca^2+^]_i_. Horizontal bars represent medians. *p < 0.05, **p < 0.01, ***p < 0.001 (Welch two-sample *t* test). **e** Current density at depolarization of a 50 mV pulse was measured and averaged for each [Ca^2+^]_i_ and then plotted against [Ca^2+^]_i_. Horizontal bars represent medians. **f** The activation time constants were obtained by fitting the outward current traces to double exponential functions. The fast time constants are plotted against membrane potential. Horizontal bars represent medians. **g** The fraction of instantaneously opening channels was calculated as the fraction of constant components of the fitting curves and is plotted against the membrane potential. Horizontal bars represent medians

The *slo-2* gene encodes a Ca^2+^-dependent K^+^ channel, which consists of an N-terminal transmembrane domain and a large C-terminal intracellular regulatory domain^20^. The H159Y mutation in *nj131* mutants is within the third helix (S3) of transmembrane domain^21^ (Supplementary Fig. 2). To examine the effects of H159Y mutation on the function of the SLO-2 channel, using the patch clamp method, we recorded whole-cell currents from HEK293T cells expressing either wild-type or H159Y mutant of SLO-2 channels. As reported previously^20^, depolarization failed to increase outward currents of wild-type and mutant channels when [Ca^2+^]_i_ was 0 but increased it when [Ca^2+^]_i_ was >0.6 μM (Fig. 2a, b). At 0.2 μM [Ca^2+^]_i_, the H159Y mutant was clearly activated in a membrane potential-dependent manner, while the wild-type was activated only slightly. The conductance–membrane potential (GV) relationship of both wild-type and mutant channels was shifted toward hyperpolarized potentials with an increase in [Ca^2+^]_i_ (Fig. 2c, d). At the same [Ca^2+^]_i_, the GV relationship of the H159Y mutant featured more hyperpolarized potential than the wild-type form, indicating that the H159Y mutant channel is more sensitive to [Ca^2+^]_i_ and to depolarization (Fig. 2c–e). In terms of activation kinetics, the time constants were smaller (Fig. 2f), and the fraction of instantaneously opening channels was larger (Fig. 2g) for the H159Y mutant channels than the wild-type at the same [Ca^2+^]_i_. Therefore, H159Y point mutation increases SLO-2 channel activity, which is consistent with the results of genetic analyses of *C. elegans* behavior (Fig. 1g, h).

### *slo-1; slo-2* double mutants are faster to change preference

**Fig. 3:**
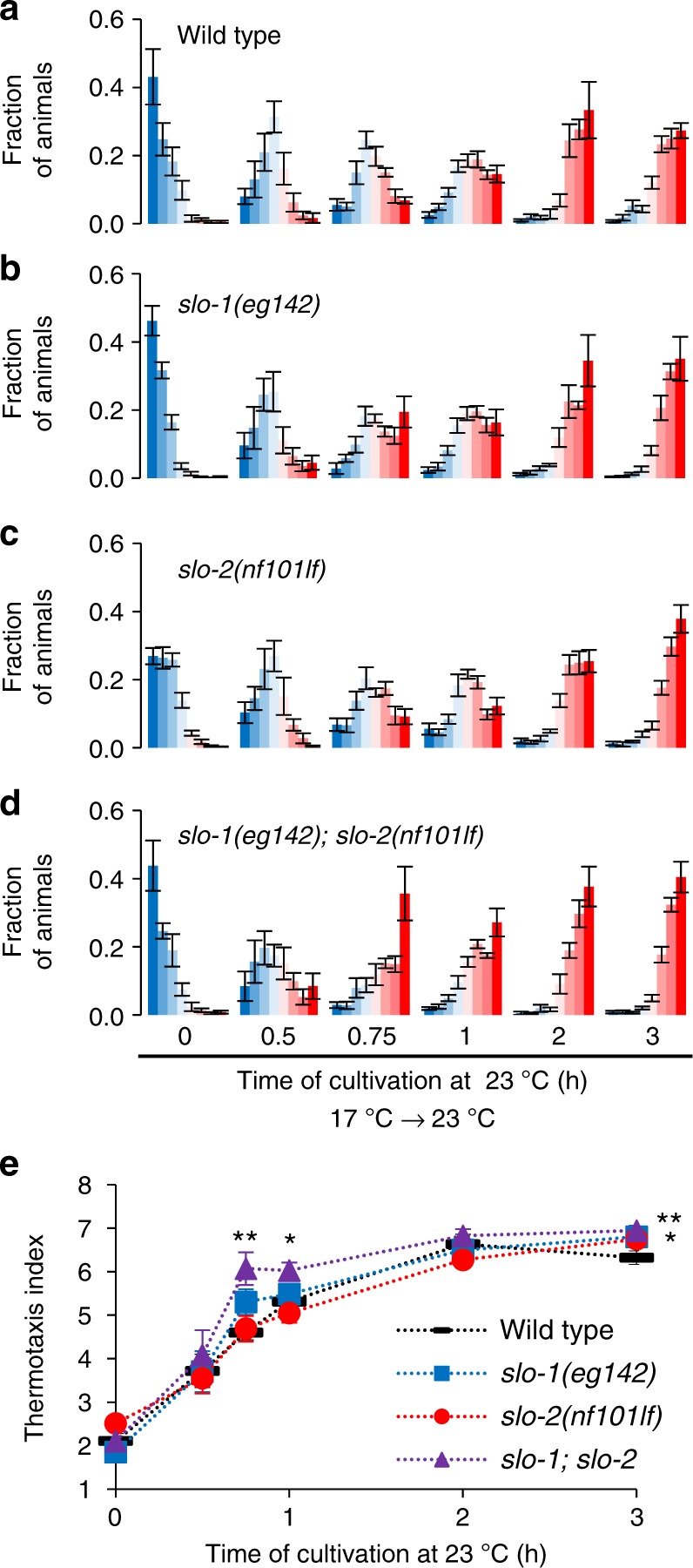
*slo-1; slo-2* double loss-of-function mutants are fast to change their behavior. **a**–**d** Wild-type (**a**), *slo-1(eg142)* (**b**), *slo-2(nf101)* (**c**), and *slo-1(eg142); slo-2(nf101)* (**d**) animals were cultivated at 17 °C for 5 days and then at 23 °C for the time indicated. The animals were then subjected to thermotaxis assay. The fractions of animals are plotted on histograms. The error bars represent the SEM. *n* = 4, 4, 6, 10, 4, 5 for wild-type animals and *n* = 6, 4, 6, 10, 4, 8 for others. **e** The means of the thermotaxis indices are plotted against time after the cultivation temperature was changed to 23 °C. The error bars represent the SEM. *p < 0.05, **p < 0.01 (Dunnett test against wild-type animals). Individual data points are shown in Supplementary Fig. 3a. See also Supplementary Figs 1, 2, and 3

Since a gf mutation in *slo-2* decelerated the transition of temperature preference, we examined whether loss of *slo-2* accelerated the transition. We revealed that the preference transition was not accelerated in *slo-2(nf101)* deletion mutants (Fig. 3c, e). Then we further examined animals lacking both SLO-2 and SLO-1. SLO-1 is structurally homologous to SLO-2 (Supplementary Fig. 2), and SLO-1 and SLO-2 channels constitute the SLO family of the K^+^ channel in *C. elegans*. In *slo-1(eg142); slo-2(nf101)* double lf mutants, preference transition was accelerated at the time points of 45 min and 1 h after an upshift in cultivation temperature (Fig. 3d, e). Another double lf mutant, *slo-1(gk262550); slo-2(ok2214)*, also showed an accelerated preference transition (Supplementary Fig. 3b and c). These results suggest that endogenous SLO K^+^ channels function redundantly^22^ in response to an upshift in cultivation temperature. Both *slo-1(lf)* and *slo-2(lf)* single mutants were normal, suggesting that both SLO-1 and SLO-2 act independently. After a downshift in cultivation temperature, *slo-1(eg142); slo-2(nf101)* double lf mutants changed their preference within the same period as the wild-type animals (Supplementary Fig. 3d), suggesting that SLO K^+^ channels regulate preference transition specifically when temperature is increased. Although vertebrate homologs of SLO-2 and SLO-1 were shown to interact^23^, *slo-2(nj131gf)* mutation decelerated preference transition even in the absence of SLO-1 (Supplementary Fig. 3e), indicating that the gf mutant form of SLO-2 can act independently of SLO-1.

### SLO K^+^ channels act in AFD thermosensory neuron

**Fig. 4:**
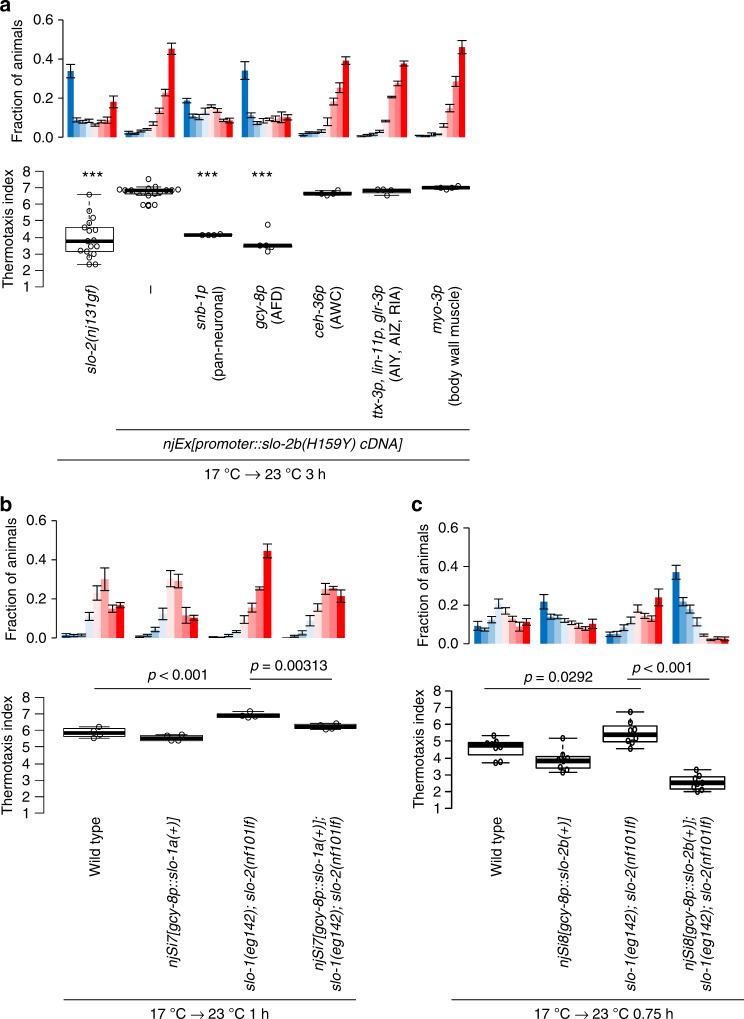
SLO K^+^ channels act in the AFD thermosensory neuron to decelerate preference transition in thermotaxis. **a** Animals expressing the H159Y mutant form of SLO-2 isoform b under the control of promoters indicated were cultivated at 17 °C for 5 days and then at 23 °C for 3 h and subjected to thermotaxis assay. The fractions of animals and thermotaxis indices are plotted. *n* = 17, 17, 4, 5, 4, 4, 4. ***p < 0.001 (Dunnett test against wild type). **b** Wild-type animals, wild-type animals that express SLO-1 in AFD after insertion of a single copy of a transgene into a genome, *slo-1(eg142); slo-2(nf101)* animals, and *slo-1(eg142); slo-2(nf101)* animals that express SLO-1 in AFD after insertion of a single copy of a transgene were cultivated at 17 °C for 5 days and then at 23 °C for 1 h. Animals were then subjected to thermotaxis assay. *n* = 4. *p* Values are indicated (Tukey-Kramer test). **c** Wild-type animals, wild-type animals that express SLO-2 in AFD after insertion of a single copy of a transgene into a genome, *slo-1(eg142); slo-2(nf101)* animals, and *slo-1(eg142); slo-2(nf101)* animals that express SLO-2 in AFD after insertion of a single copy of a transgene were cultivated at 17 °C for 5 days and then at 23 °C for 45 min. Animals were then subjected to thermotaxis assay. *n* = 8. *p* Values are indicated (Tukey-Kramer test). See also Supplementary Fig. 4

Since *slo-2* is expressed broadly in neurons and muscles (Supplementary Fig. 4a)^20^, we aimed to identify the cells in which SLO K^+^ channels act during temperature preference transition. Expression of the H159Y gf form of SLO-2 pan-neuronally or specifically in AFD thermosensory neuron phenocopied *slo-2(nj131gf)* mutants, while expression in the AWC chemosensory and thermosensory neuron, in interneurons involved in the regulation of thermotaxis, or in body wall muscles did not (Fig. 4a). These results suggest that SLO-2 acts in AFD to decelerate preference transition.

The abilities of the wild-type and H159Y mutant form of SLO-2 to decelerate preference transition were compared by expressing various concentrations of each form in AFD. Injection of a plasmid containing the H159Y form at 1 ng/μl decelerated the preference transition more than injection of the wild-type at 20 ng/μl, suggesting that the H159Y form is far more potent in decelerating the preference transition (Supplementary Fig. 4b and c). These results are consistent with electrophysiological experiments revealing that mutant channels engage in higher levels of activity (Fig. 2c–e).

We next examined whether the accelerated preference transition in *slo-1; slo-2* double lf mutants was caused by the loss of SLO channels in AFD. Expression of SLO-1 or SLO-2 in AFD reversed the accelerated preference transition in *slo-1; slo-2* mutants (Fig. 4b, c), indicating that loss of SLO-1 and SLO-2 causes accelerated preference transition and that SLO-1 and SLO-2 act in AFD. Together with the results of the cell-specific expression experiments (Fig. 4a), it can be concluded that SLO K^+^ channels act in AFD to decelerate preference transition.

### CNG-3 CNG channel functions with SLO-2

**Fig. 5:**
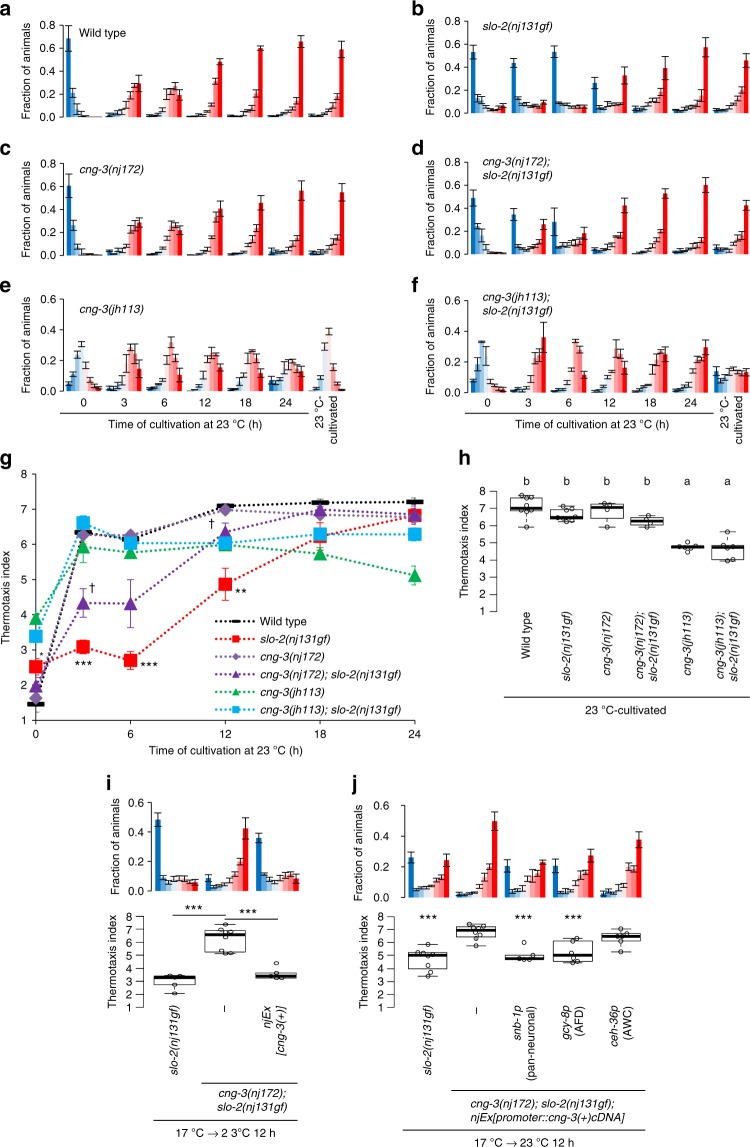
*cng-3* loss-of-function suppresses decelerated preference transition in *slo-2(nj131gf)* mutants. **a**–**f** Wild-type (**a**, *n* = 3, 3, 5, 3, 3, 6, 8), *slo-2(nj131gf)* (**b**, *n* = 3, 9, 5, 5, 3, 3, 8), *cng-3(nj172)* (**c**, *n* = 3, 3, 3, 3, 3, 5, 4)*, cng-3(nj172); slo-2(nj131gf)* (**d**, *n* = 3, 9, 5, 5, 3, 3, 4)*, cng-3(jh113)* (**e**, *n* = 5, 3, 3, 3, 3, 5, 6), and *cng-3(jh113); slo-2(nj131gf)* (**f**, *n* = 3, 3, 3, 3, 3, 5, 6) animals were cultivated at 17 °C for 5 days and then at 23 °C for the time indicated or constantly at 23 °C for 3 days. Animals were then subjected to thermotaxis assay. The fractions of animals are plotted. **g** The means of the thermotaxis indices at each time point (**a**–**f**) are plotted against time after cultivation temperature was changed to 23 °C. The error bars represent the SEM. *p < 0.05, **p < 0.01, ***p < 0.001 between wild-type and *slo-2(nj131gf)* animals, and ^†^*p* < 0.05 between *slo-2(nj131gf)* and *cng-3(nj172); slo-2(nj131gf)* animals (Tukey–Kramer test). **h** The thermotaxis indices for animals cultivated constantly at 23 °C in **a**–**f** are shown. The indices of strains marked with distinct alphabets differ significantly (*p* < 0.001) according to Tukey–Kramer test. **i** Genomic PCR fragments covering the *cng-3* locus were injected into *cng-3(nj172); slo-2(nj131gf)* animals. Animals were cultivated first at 17 °C, then at 23 °C for the time indicated, and then subjected to thermotaxis assay. *n* = 5, 8, 5. ***p < 0.001 (Tukey–Kramer test). **j** *cng-3(nj172); slo-2(nj131gf)* animals expressing *cng-3* under control of the indicated promoters were cultivated at 17 °C for 5 days, then at 23 °C for 12 h, and then subjected to thermotaxis assay. *n* = 8, 8, 6, 6, 5. ***p < 0.001 (Dunnett test against *cng-3(nj172); slo-2(nj131gf)* animals). See also Supplementary Figs. 5 and 6

To investigate how SLO K^+^ channels decelerate preference transition, we performed a genetic screen for the suppressors of *slo-2(nj131gf)* mutants to isolate molecules that functionally interact with SLO-2 (Supplementary Fig. 5a). Of the mutagenized *slo-2(nj131gf)* mutant animals, we isolated *nj172* mutation, which partially suppressed the Slw phenotype of *slo-2(nj131gf)* (Fig. 5a, b, d, g). When cultivated at a constant temperature of 17 °C, both *nj172; slo-2(nj131gf)* double mutants and wild-type animals migrated to a cold region (Fig. 5d, 0 h), suggesting that *nj172* does not make animals simply thermophilic but suppresses the Slw phenotype in *slo-2(nj131gf)* animals.

SNP mapping and subsequent rescue experiments showed that *nj172* is an allele of *cng-3* (Fig. 5i, Supplementary Fig. 5h and i). The *cng-3* gene encodes a subunit of the CNG cation channel. Whole-genome sequencing revealed that *nj172* mutants carried a missense mutation in the *cng-3* gene, which alters methionine residue 467 to isoleucine (M467I) within a conserved C-terminal cyclic nucleotide-binding domain of the gene product^24^. When cultivated constantly at 23 °C, *nj172; slo-2(nj131gf)* animals expressing *cng-3* migrated to a warm region similarly to wild-type animals (Supplementary Fig. 5i), suggesting that *cng-3* expression does not make animals simply cryophilic but cancels the suppression of *slo-2(nj131gf)* by *cng-3(nj172)*.

We also characterized *cng-3(jh113)* deletion mutants. When first cultivated at 17 °C and then at 23 °C for 3 h, *cng-3(jh113); slo-2(nj131gf)* animals migrated toward the warm region on a thermal gradient (Fig. 5f, g), suggesting that *cng-3(jh113)* deletion mutation also suppresses the Slw phenotype of *slo-2(nj131gf)* animals. The extent of suppression seemed stronger than that of *cng-3(nj172)* mutation, suggesting that *cng-3(nj172)* is a reduction-of-function allele. When cultivated constantly at 17 °C or 23 °C, both *cng-3(jh113); slo-2(nj131gf)* and *cng-3(jh113)* mutants did not show a clear preference for a cultivation temperature (Fig. 5e–h)^25^. The abnormal thermotaxis in *cng-3(jh113)* mutants cultivated constantly at 17 °C or 23 °C were rescued by expressing CNG-3 in AFD (Supplementary Fig. 6a and b). These results indicate that CNG-3 is necessary for thermotaxis after cultivation under constant temperature but unnecessary for acquisition of a new preference after a shift in cultivation temperature. Other CNG subunits, TAX-2 and TAX-4, are critical for thermosensation; animals lacking either TAX-2 or TAX-4 were completely athermotactic, regardless of whether cultivation temperature was shifted (Supplementary Fig. 6c).

It was reported that CNG-3 is expressed in sensory neurons, including AFD^26,27^. To determine the site(s) at which CNG-3 acts, we cell-specifically expressed CNG-3 in *cng-3(nj172); slo-2(nj131gf)* mutants. Expression of CNG-3 in AFD but not in AWC canceled suppression by *cng-3(nj172)* (Fig. 5j), suggesting that, like SLO channels, CNG-3 functions in AFD (Fig. 4).

### SLO and CNG channels slowed down AFD adaptation

**Fig. 6:**
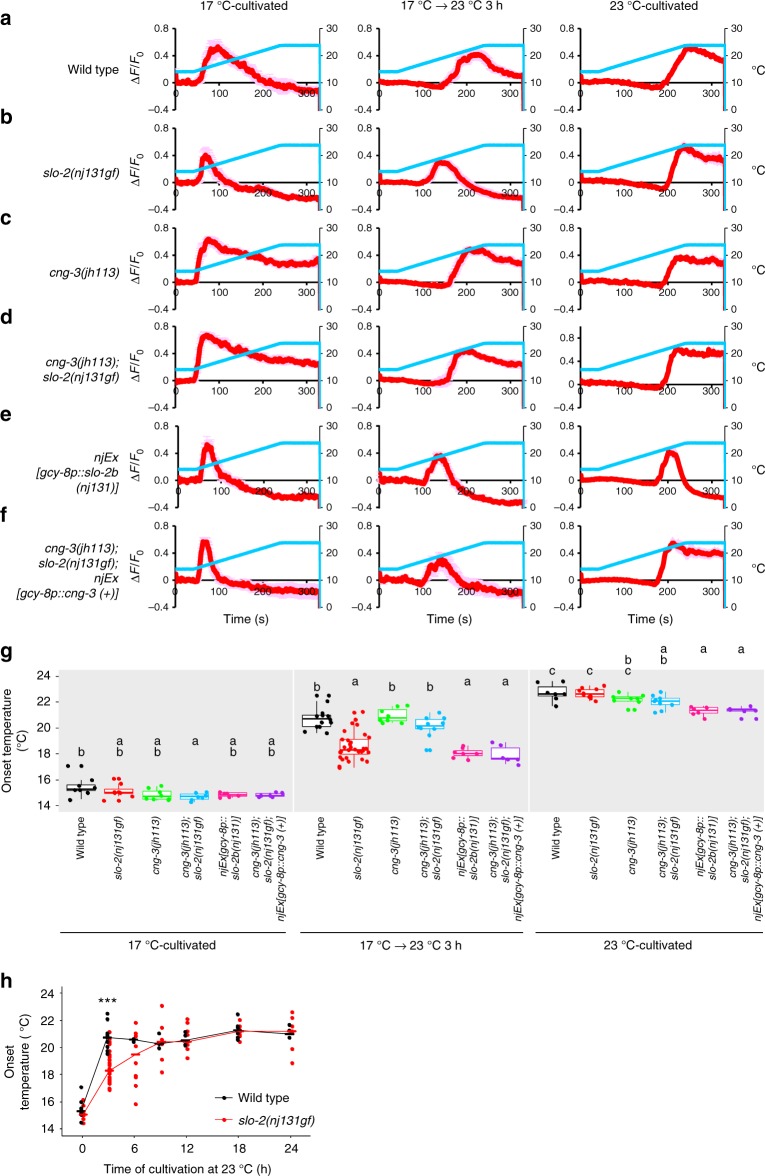
SLO K^+^ channels slow down AFD adaptation. **a**–**f** Wild-type (**a**, *n* = 8, 13, 7), *slo-2(nj131gf)* (**b**, *n* = 8, 31, 10), *cng-3(jh113)* (**c**, *n* = 8, 8, 8), and *cng-3(jh113); slo-2(nj131gf)* (**d**, *n* = 7, 10, 9) animals; animals expressing SLO-2b(H159Y) in AFD (**e**, *n* = 6, 7, 6); and *cng-3(jh113); slo-2(nj131gf)* animals expressing CNG-3 in AFD (**f**, *n* = 6, 6, 6) that expressed R-CaMP2 in AFD were cultivated at 17 °C for 5 days, at 17 °C for 5 days and then at 23 °C for 3 h or at 23 °C for 3 days. Animals were then subjected to Ca^2+^ imaging analysis. The animals were initially kept at 14 °C for 40 s and then subjected to a linear temperature rise to 24 °C over 200 s followed by 90 s of incubation at 24 °C. Temperature stimuli and changes in fluorescence intensity are indicated with blue and red lines, respectively. Data were collected from distinct animals. Pale red shadow represents the SEM. **g** The onset temperatures at which the AFD response reached half of the maximum during the above experiment (**a**–**f**) are plotted. The onset temperatures of strains marked with distinct alphabets differ significantly (*p* < 0.05) according to Tukey–Kramer test under the same cultivation conditions. **h** Wild-type and *slo-2(nj131gf)* animals that express R-CaMP2 in AFD were cultivated at 17 °C for 5 days and then at 23 °C for the time indicated. Animals were then subjected to Ca^2+^ imaging with the same temperature increase stimulus as in **a**–**f**. Onset temperature is plotted, and medians are indicated with horizontal bars. Data at 0 and 3 h are identical to those in **a**, **b**. *n* = 3, 3, 4, 6, 4 for wild-type and *n* = 10, 8, 9, 8, 7 for *slo-2(nj131gf)* animals at each time point (6, 9, 12, 18, and 24 h). ***p < 0.001 (Welch two-sample *t* test between wild-type and *slo-2(nj131gf)* animals)

Since SLO-2 was shown to act in the AFD thermosensory neuron to decelerate temperature preference transition, we next examined whether SLO-2 acts upstream of Ca^2+^ responses in AFD using Ca^2+^ imaging. AFD responds to rises in temperature above the past cultivation temperature^7,8^. In wild-type animals cultivated constantly at 17 °C, AFD increased the Ca^2+^ level at approximately 15 °C in response to a temperature increase from 14 °C to 24 °C (Fig. 6a, g). In wild-type animals, the temperature leading to onset of AFD responses when cultivated constantly at 23 °C was around 23 °C, but the onset temperature of AFD response when cultivated first at 17 °C and then at 23 °C for 3 h was around 21 °C. Although we did not detect any obvious abnormality in the onset temperature of *slo-2(nj131gf)* animals cultivated constantly at 17 °C or at 23 °C, the onset temperature in *slo-2(nj131gf)* animals cultivated first at 17 °C and then at 23 °C for 3 h was lower than in wild-type animals (Fig. 6b, g). When cultivated longer at 23 °C, the onset temperature for *slo-2(nj131gf)* animals gradually became comparable to that for wild-type animals (Fig. 6h). These results suggest that *slo-2(nj131gf)* mutation slowed down transition of the AFD dynamic range (i.e., AFD adaptation). AFD adaptation preceded behavioral transition in both wild-type animals and *slo-2(nj131gf)* mutants (Figs. 1e and 6h). These results indicate that a mechanism is acting either downstream of the Ca^2+^ influx in AFD or in downstream neural circuits, which may explain that the transition of the AFD dynamic range is transformed onto the behavior with delay. Moreover, expression of the gf form of SLO-2 specifically in AFD neurons lowered the onset temperature for animals cultivated first at 17 °C and then at 23 °C for 3 h (Fig. 6e, g), suggesting that enhanced SLO-2 currents in AFD slow down AFD adaptation cell-autonomously.

Since the Slw phenotype in *slo-2(nj131gf)* mutants was suppressed by *cng-3* mutation, we next examined whether the slowed AFD adaptation in *slo-2(nj131gf)* mutants after an upshift in cultivation temperature was also suppressed by *cng-3* mutation. When cultivated first at 17 °C and then at 23 °C for 3 h, *cng-3(jh113)* suppressed the lowered onset temperature of AFD in *slo-2(nj131gf)* animals to a level comparable to that of wild-type animals (Fig. 6d, g). Moreover, AFD-specific expression of CNG-3 in *cng-3(jh113); slo-2(nj131gf)* double mutants canceled the suppression of decelerated adaptation by *cng-3* lf mutation (Fig. 6f, g). These results suggest that CNG-3 functionally interacts with SLO-2 in AFD to slow down AFD adaptation and thereby to decelerate behavioral transition. Although *cng-3(jh113)* mutants showed aberrant thermotaxis behavior when cultivated constantly at 17 °C or at 23 °C (Fig. 5e), AFD in *cng-3(jh113)* mutants responded to a temperature increase at around the cultivation temperature, like the wild-type animals, except for a slower decrease in the level of Ca^2+^ (Fig. 6c). This is in a sharp contrast to *tax-2* or *tax-4* mutants, in which AFD is totally inactive^8,9^.

**Fig. 7:**
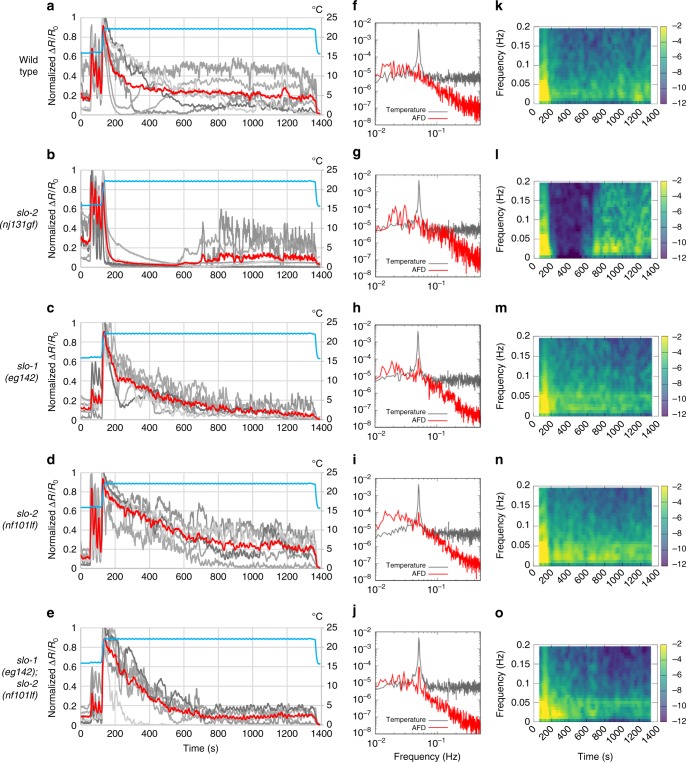
Endogenous SLO K^+^ channels slow down AFD adaptation. **a**–**e** Wild-type (**a**), *slo-2(nj131gf)* (**b**), *slo-1(eg142)* (**c**), *slo-2(nf101)* (**d**), and *slo-1(eg142); slo-2(nf101)* (**e**) animals that express GCaMP3 and tagRFP in AFD were cultivated at 17 °C for 5 days and subjected to Ca^2+^ imaging analysis with a temperature stimulus involving 20 s (0.05 Hz) oscillations around 17 °C followed by a temperature upshift to 23 °C and oscillations around 23 °C. The intensity of green fluorescence was divided by that of red fluorescence, and the ratio was normalized to a range between 0 and 1 and plotted against time. Gray and red lines indicate traces from individual animals and the average, respectively. Data were collected from distinct animals (*n* = 6). **f**–**j** The Fourier transform was computed with the Hanning window on the temperature program and ratio of fluorescence intensity between 401 and 1390 s for each animal in **a**–**e**. Averaged power spectra are plotted against frequency. **k**–**o** Time evolutions of averaged spectrograms are plotted in a color map against the centric time of each segment. Trends detected by Butterworth filter were removed from the ratio of fluorescence intensity of each animal in **a**–**e**. The resulting signals were divided into segments of 128 s, and the Fourier transform was separately computed for each segment

We next examined how endogenous SLO channels affect the timing of changes in the AFD dynamic range. The dynamic range of AFD changes within a few hours after a shift in cultivation temperature^16,28^, whereas AFD is irresponsive to an increase in temperature beyond the temperature 5–6 °C higher than the cultivation temperature^8^. Thus animals cultivated at 17 °C were investigated when their AFD regained the responsiveness after an upshift in cultivation temperature. To do so, we used a temperature program that produced short-term temperature oscillations (20 s, 0.05 Hz) around 17 °C followed by a shift to 23 °C, which mimicked an upshift in cultivation temperature, and long-term oscillations around 23 °C (Fig. 7a–e). In wild-type animals, AFD exhibited phase-locked responses to oscillations around 17 °C, a large response to a temperature upshift and gradual Ca^2+^ decrease, and then faintly regained phase-locked responses to oscillations around 23 °C 20 min after the upshift (Fig. 7a). In contrast, in *slo-2(nj131gf)* animals, AFD showed a sharp decrease in Ca^2+^ level and profound silencing after the temperature upshift and did not regain the ability for phase-locked response in 20 min (Fig. 7b). On the other hand, in *slo-1; slo-2* double lf mutants, AFD showed clear phase-locked responses to oscillations from an earlier time point than wild-type animals (Fig. 7e). Use of the Fourier transform to examine the AFD responses further supported these observations (Fig. 7f–j). First, Fourier transform of the sequence of temperature revealed a clear peak at 0.05 Hz. The peak at 0.05 Hz in the AFD responses of *slo-1; slo-2* double lf mutants was much more explicit than that of wild-type animals (Fig. 7f, j). In time courses of the Fourier transform, the Fourier components of AFD response other than those at or near 0.05 Hz disappeared earlier in *slo-1; slo-2* double lf mutants than in wild-type animals (Fig. 7k, o). Taken together, these results further support that SLO K^+^ channels slow down AFD adaptation after an upshift in cultivation temperature.

### Epilepsy-related mutations potentiate SLO-2

Early-onset forms of epilepsy such as malignant migrating partial seizures of infancy and autosomal-dominant nocturnal frontal lobe epilepsy can be caused by gf mutations in the *slo-2* human homolog *Slack/KCNT1*^17,18^, which encodes a Na^+^-gated K^+^ channel mainly expressed in the brains^29^. Epilepsy-related mutant forms of the Slack channel feature enhanced channel activity^17,30–32^. Analogously, the H159Y mutant form of *C. elegans* SLO-2 isolated in this study featured higher channel activity (Fig. 2), suggesting that both human epilepsy and decelerated temperature preference transition in thermotaxis are caused by excess K^+^ efflux through SLO-2 channels. Therefore, we tested whether introduction of epilepsy-related mutations in *C. elegans* SLO-2 (Supplementary Figs. 2 and 7) could phenocopy the gf nature of H159Y for decelerating the preference transition in thermotaxis.

**Fig. 8:**
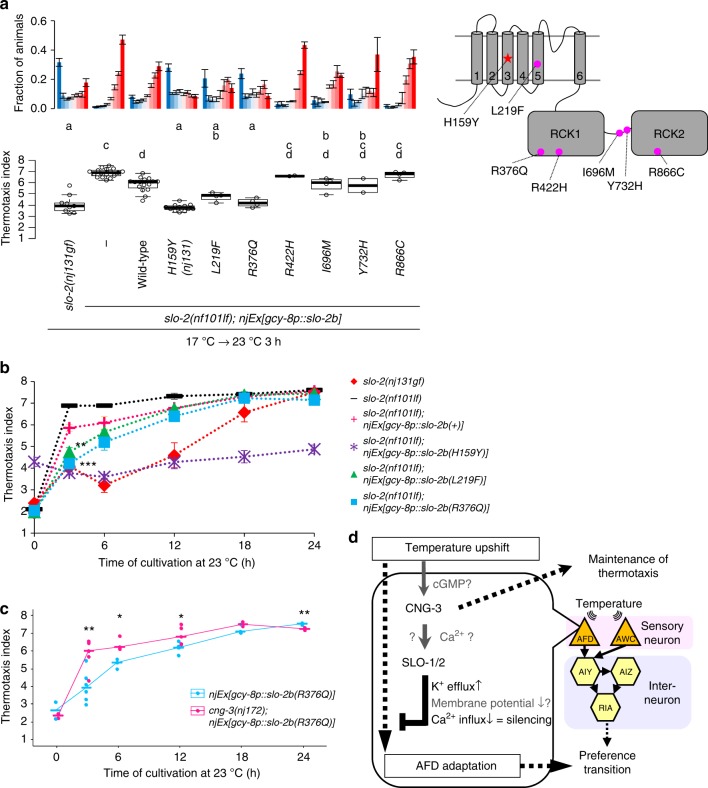
Epilepsy-related mutations potentiate SLO-2. **a** *slo-2(nf101)* animals expressing either wild-type or the indicated mutant form of SLO-2b in AFD were cultivated at 17 °C for 5 days and then at 23 °C for 3 h and then subjected to thermotaxis assay. Fractions of animals are plotted (upper). *n* = 9, 17, 16, 12, 4, 4, 2, 4, 2, 4. Thermotaxis indices of strains marked with distinct alphabets differ significantly (*p* < 0.05) according to Tukey–Kramer test (lower). **b** *slo-2(nf101)* animals expressing either wild-type or the indicated mutant form of SLO-2b in AFD were cultivated at 17 °C for 5 days and then at 23 °C for the indicated time points. The animals were then subjected to thermotaxis assay. The means of thermotaxis indices are shown. The error bars represent the SEM. Data at 3 h are identical to those in **a**. **p < 0.01, ***p < 0.001 (Tukey–Kramer test, compared with animals expressing SLO-2b(+)). The fractions of animals and individual indices at each time point are shown in Supplementary Fig. 7. **c** Animals expressing SLO-2b(R376Q) in AFD with either a wild-type or *cng-3(nj172)* background were cultivated at 17 °C for 5 days, then at 23 °C for the indicated time points, and then subjected to thermotaxis assay. Thermotaxis indices at each time point are shown. The horizontal bars represent the medians. *n* = 2, 6, 3, 5, 3, 3 for each time point. *p < 0.05, **p < 0.01 (Welch two-sample *t* test between two strains at each time point). The fractions of animals are shown in Supplementary Fig. 7. **d** A model for regulation of the timing of preference transition in thermotaxis is shown. SLO K^+^ channels and CNG-3 generate latency for preference transition in thermotaxis after an upshift in cultivation temperature by acting in AFD to slow down the AFD adaptation to new temperature. See also Supplementary Figs. 2, 3, 7, and 8

We introduced each of six epilepsy-related mutations into *C. elegans slo-2* DNA and injected each DNA into *slo-2(nf101)* deletion mutants. Animals expressing wild-type or mutant SLO-2 in AFD were cultivated first at 17 °C, then at 23 °C for 3 h, and then were subjected to thermotaxis assay. Expression of two of six epilepsy-related mutant forms of SLO-2 more greatly decelerated preference transition than did expression of wild-type SLO-2 (Fig. 8a, b). These results imply that, although slow preference transition in *C. elegans* thermotaxis and early-onset epilepsy in humans are macroscopically divergent, both events result from common cellular processes initiated by a gf mutation in the SLO-2 channel. Excessive activity of SLO channels and resulting excessive repolarization after depolarization either in AFD thermosensory neurons in *C. elegans* or in neurons in the human brains seem to cause the respective event. These results also indicated that these two mutations potentiate SLO-2/Slack channels, regardless of their differences such as gating by Ca^2+^ or Na^+^. We also found that *cng-3* mutation suppressed deceleration of preference transition caused by the expression of SLO-2(R376Q) in AFD (Fig. 8c), implying that inhibition of CNG channels might attenuate epilepsy symptoms. It is of clinical interest to examine whether *Slack(gf)* rodent models show epilepsy-related phenotypes and whether CNG inhibitors attenuate those phenotypes.

## Discussion

In this study, we demonstrated that, upon an upshift in cultivation temperature, SLO K^+^ channels function with the CNG-3 channel in the AFD thermosensory neuron to slow down the transition of the AFD dynamic range and thereby generate latency for transition of the temperature preference. An upshift in the temperature results in a large influx of Ca^2+^ into AFD, and consequently K^+^ efflux through SLO channels repolarizes the membrane potential and decreases [Ca^2+^]_i_, which antagonize premature AFD adaptation (Fig. 8d). Although the expression levels of AFD-specific guanylyl cyclases are shown to increase when cultivation temperature is increased^28^, this induction was unaffected by *slo-2(nj131gf)* mutation (Supplementary Fig. 8a–c). We also found a molecular and physiological link between early-onset epilepsy and preference transition in thermotaxis, providing a model system that can be used to understand the pathogenesis of this type of epilepsy and to screen for drugs.

Although CNG channels are generally thought to depolarize the membrane potential^24^, our results implied that CNG-3 acts with SLO K^+^ channels to hyperpolarize AFD (Fig. 5). Considering that SLO-1 and SLO-2 of *C. elegans* are both gated by Ca^2+^^33^ and that CNG channels are generally permeable to Ca^2+^^24^, it is reasonable to hypothesize that SLO and CNG channels are co-localized in a limited region in which local Ca^2+^ influx through CNG channels, including CNG-3, activates SLO K^+^ channels.

While the onset temperature of AFD in *slo-2(nj131gf)* animals cultivated at 17 °C was comparable with that in wild-type animals, the Ca^2+^ level decreased sharply beneath the base line in *slo-2(nj131gf)* animals (Fig. 6b). This result is consistent with the important roles of SLO-2 in post-excitatory repolarization of the membrane potential in body wall muscles^29,34,35^ and in motor neurons^36^ in *C. elegans* and in mammalian neurons^37,38^. This altered AFD response accompanied with the diffused distribution during thermotaxis behavior of animals cultivated at 17 °C in both *slo-2(nj131gf)* animals (Figs. 6b, 1d) and animals with AFD-specific SLO-2 expression (Fig. 6e and Supplementary Fig. 7f, 0h and 7i). In addition, both the altered AFD response and the diffused distribution of the animals were suppressed by *cng-3* mutation (Figs. 6d and 5d). Thus the sharp decrease in Ca^2+^ in AFD may result in insufficient navigation during thermotaxis.

Although a number of studies have focused on alteration of the relationships between unconditional stimuli (US) and conditional stimuli (CS)^39^, our study focused on CS transition. *C. elegans* associate CS such as temperature or chemicals with preferable US such as the existence of food, and the conditioned animals are attracted to the CS. Similarly, they can also associate CS with unpreferable US and no longer be attracted to the CS or even avoid it. For instance, the role of the insulin–phosphoinositide-3 kinase–Akt pathway was intensively studied during association between CS with starvation as a negative US^40–42^. The mechanisms underlying decay of the US–CS association have also been reported^43–45^. In this study, we performed a forward genetic screen that focused on CS transition and newly identified molecules that do not have any reported roles in learning. *dgk-3* mutants were previously reported to be slow to change temperature preference in thermotaxis after cultivation temperature was shifted^16^. However, *dgk-3* mutants in our experimental set-up were simply cryophilic. In this study, our results suggested that the SLO and CNG channels act together to modulate transition of the dynamic range of a sensory neuron that can encode the CS.

Unveiling the mechanisms underlying short- and long-term memory (STM and LTM, respectively) and consolidation of STM into LTM is an important issue in neuroscience. The role of the cAMP–protein kinase A–CREB pathway in consolidation of Pavlovian conditioning is conserved among Aplysia, flies, and mammals. Our results may provide a foundation for further genetic research to reveal the mechanisms underlying consolidation. Since the original report on thermotaxis^5^, it has been known that temperature preference reaches a local maximum 2–3 h after a shift in cultivation temperature, is slightly weakened, and then reaches a plateau (Fig. 1e). We found that *slo-2(nj131gf)* animals were slow to change their behavior, whereas *cng-3(jh113)* animals could transiently acquire an ability to perform thermotaxis immediately after a shift in cultivation temperature but gradually lost the ability when cultivated for longer at the new temperature. One possibility is that two different components, one of which is fast but transient and the other of which is delayed but persistent, might help shape the transition of temperature preference (Supplementary Fig. 8d). Our results suggested that SLO and CNG channels suppress the fast component and that the delayed component is dependent on CNG-3 but not on SLO channels, since *slo-1; slo-2* double lf mutants exhibited normal thermotaxis when cultivated for a long time at a certain temperature. Revealing the mechanisms by which each component is regulated, through screening for suppressors for *cng-3* mutants or analyzing animals in a transient state of consolidation, might lead to a comprehensive understanding of consolidation of STM into LTM.

Vertebrate CNG channels, which are essential for the functions of sensory neurons such as rod and cone photoreceptors and olfactory receptor neurons, are also expressed in the central nervous system and play versatile roles^46^. CNG channels with different subunit composition have different properties, such as sensitivity to cNMP and ion permeability, and function in different cell types^24^. The results of this study and others provide clues that improve our understanding of how CNG channels with distinct subunit compositions contribute to different cellular processes. Among the CNG channels in *C. elegans*, TAX-2 and TAX-4 are essential for thermotaxis as well as for chemotaxis^14,15^, but CNG-3 seems to play an auxiliary role^47^. Although an α subunit TAX-4 can form a functional homo-tetramer that is far more sensitive to cGMP than the heteromer consisting of TAX-4 and a β subunit TAX-2^48^ and TAX-2 cannot form a functional homomer by itself, TAX-2 is essential for thermosensation and chemosensation, indicating that heteromer-specific functions exist. CNG-3 was suggested to form a hetero-tetramer with TAX-4 and TAX-2 in AWC^47^, implying that a similar heteromer also forms in AFD. Our results suggest that CNG-3 is necessary for steady, but not for transient, thermotaxis. Thus investigating how CNG channels with different compositions function in specific behavioral or cellular contexts in combination with the analysis of their electrophysiological properties would provide further understanding of how CNG channels function in the nervous system.

This study, focusing on a transient state of decision making, not only provided scientific insights into the mechanisms that determine the timing of decision making but also revealed possible clinical applications that may contribute to the treatment of early-onset epilepsy. Our results suggested that the preference transition in thermotaxis might be useful as a model system for early-onset epilepsy caused by Slack/KCNT1 mutations. Given that quinidine, which blocks the Slack channel^49,50^, was not effective in every case of Slack/KCNT1-related epilepsy^51–53^ and that quinidine blocks other ion channels and may cause cardiac toxicity^50^, screening for drugs that specifically affect Slack using *C. elegans* would be valuable. Since Slack is expressed in cardiomyocytes^29^, identification of molecules that functionally interact with Slack only in neurons by screening suppressors for *slo-2(nj131gf)* animals would identify better drug targets.

## Methods

### Experimental model and subject details

*C. elegans* strains were maintained as described^54^. Briefly, animals were cultivated on nematode growth medium (NGM) plates seeded with an *Escherichia coli* OP50 strain (Caenorhabditis Genetics Center (CGC), Twin Cities, MN, USA). N2 (Bristol) was used as the wild-type strain unless otherwise indicated. Transgenic lines were generated as described^55^. Briefly, plasmid DNA or PCR fragments were directly injected into the hermaphrodite gonad. Strains used in this study are listed in Supplementary Table 2.

### Behavioral assays

Population thermotaxis assays were performed as described previously^56^. Briefly, 50–250 animals cultivated at 17 °C or 23 °C or subjected to a shift in cultivation temperature were placed at the center of the assay plates without food and with a temperature gradient of 17–23 °C. The animals were allowed to freely move for 60 min. The assay plate was divided into eight sections along the temperature gradient, and the number of adult animals in each section was scored. The ratio of animals in each section was plotted on histograms. Thermotaxis indices were calculated as shown below:

$$\frac{{\mathop {\sum }\nolimits_{i = 1}^8 i \bullet N_i}}{N}$$

where *N*_*i*_: number of animals in each section *i* (*i* = 1–8) and *N*: total number of animals on the test plate.

### Forward genetic screen for Slw mutants

For mutagenesis, wild-type animals were treated with 50 mM ethyl methanesulfonate (EMS; Nacalai, Kyoto, Japan) for 4 h at room temperature. The F1 generation of the mutagenized animals was cultivated at 17 °C for 5 days and was allowed to self-fertilize and give rise to the F2 generation. F2 animals were cultivated at 23 °C for 3 h and then put on a thermal gradient for thermotaxis assay. Most animals migrated to the warm region of the thermal gradient. Animals that still migrated to the cold region and thus were putatively slow to change behavior, were isolated. To discriminate between animals that are slow to change behavior and those that constitutively prefer cold temperature, the strains isolated above were cultivated constantly at 23 °C and then subjected to thermotaxis assay. Animals that migrated to the cold region when cultivated at 17 °C and then at 23 °C for 3 h but migrated to the warm region when cultivated constantly at 23 °C were considered to be slow to change behavior.

### Suppressor screen against *slo-2(nj131gf)*

As described above, *slo-2(nj131gf)* animals were mutagenized with EMS. Mutagenized animals were cultivated at 17 °C for 5 days and at 23 °C for 3 h and then were subjected to thermotaxis assay as described above. Most animals still migrated to the cold region of the thermal gradient. Animals that migrated to the warm region like the wild-type animals were isolated.

### Mapping of *nj131* and *nj172*

We crossed *nj131* animals with a wild-type polymorphic CB4858 strain^57^. Since the Slw phenotype of *nj131* was inherited semidominantly, we isolated F2 animals that exhibited Slw or wild-type phenotypes and identified crossover sites as described^58^. We mapped *nj131* to a 1.3-Mb interval between nucleotides 11,136,567 and 12,419,537 on linkage group (LG) *X*.

To map *nj172*, we first crossed *slo-2(nj131gf)* animals with CB4858 four times to obtain a strain in which the *slo-2(nj131gf)* allele exists on the CB4858 genome (IK2092). We then crossed *nj172; slo-2(nj131gf)* with IK2092, isolated F2 animals in which the Slw phenotype was suppressed and identified crossover sites. We mapped *nj172* to a 1.1-Mb interval between nucleotides 8,723,437 and 9,847,895 on LG *IV*.

### Whole-genome sequencing

Genomic DNA was purified with the Puregene Core Kit A (Qiagen, Hilden, Germany). The genome was sequenced with MiSeq (Illumina, San Diego, CA, USA), and the sequence was analyzed with CLC Genomics Workbench (CLC Bio, Aarhus, Denmark).

### Plasmids

pOX nSlo2 was a gift from Larry Salkoff (Addgene plasmid #16202)^20^. A DNA clone including a part of *cng-3* cDNA (yk348a9) was provided by Yuji Kohara. To generate plasmids to cell-specifically express *slo-2* or *cng-3*, we fused promoter sequences of *gcy-8*, *ceh-36*, *ttx-3*, *lin-11*, *glr-3*, or *myo-3*; the cDNA of *slo-2* or *cng-3*; and the *unc-54* 3'UTR sequence by MultiSite Gateway Technology (Thermo Fisher Scientific, Waltham, MA, USA). To generate plasmids to express SLO-2b in HEK293T cells, *slo-2b* cDNA was inserted into a pCAG vector. Details regarding the plasmid constructs can be obtained from the authors. *Peft-3::Cas9-SV40 NLS::tbb-2 3'-UTR* (Addgene plasmid #46168) and *PU6::unc-119_sgRNA* (#46169) were gifts from John Calarco^59^. The *unc-119* single-guide RNA (sgRNA) sequence in *PU6::unc-119_sgRNA* was replaced with the sequences of sgRNAs for *unc-22*, *slo-2* #1, and *slo-2* #4, which were GAACCCGTTGCCGAATACAC(AGG)^60^, GAAATACCTGGTTTTCGGGG(TGG), and GCACAATGCACGATGCAAGG(CGG), respectively. Erik Jorgensen gifted pCFJ150 *pDESTttTi5605[R4-R3]* (Addgene plasmid #19329), pCFJ601 *Peft-3::Mos1 transposase* (#348874), pMA122 *Phsp::peel-1* (#348873), pCFJ90 *myo-2p::mCherry* (#19327), pCFJ104 *myo-3::mCherry* (#19328), and pGH8 *rab-3p::mCherry* (#19359)^61,62^. A plasmid containing *slo-1* cDNA was a gift from Jonathan T. Pierce^63^. The *gcy-8* promoter sequence, *slo-1* cDNA, and *unc-54* 3'UTR sequence were fused into pCFJ150 to make pIA109. Plasmids used in this study are listed in Supplementary Table 3.

### Knockout of *slo-2* by CRISPR/Cas9

The *slo-2* gene was knocked out by CRISPR/Cas9 as described^60,64^. First, *slo-2(nj131gf)* animals were injected with *Peft-3::Cas9-SV40 NLS::tbb-2 3'-UTR*, pRF4 *rol-6(su1006, gf)*, pSN680 *unc-22* sgRNA, pIA009 *slo-2* sgRNA #1, and pIA054 *slo-2* sgRNA #4. Then *unc-22/+* and *unc-22/unc-22* F1 twitcher animals were subjected to genotyping for the *slo-2* gene locus. Although deletion between the sites of sgRNA #1 and #4 was not detected, small indels around the sgRNA #1 site were detected by polyacrylamide gel electrophoresis following PCR that amplified 60 bp region including the site of sgRNA#1 with primers TCGTGATTTCTTGGAAGAATTCT and tggtgtgttactgaaatacCTGG. A strain that homozygously carried a small deletion at the *slo-2* locus was isolated from the progeny of a *unc-22/+* animal with a small deletion. Sequencing of the genomic *slo-2* locus revealed that, in this strain, two nucleotides in the seventh exon are deleted, causing a frame shift after His344. No indel was found adjacent to the site of sgRNA #4. Then *unc-22* mutation was knocked out by selecting non-twitcher animals.

### Single-copy insertion by MosSCI

A single copy of *gcy-8p::slo-1a(+)* or *gcy-8p::slo-2b(+)* was inserted into a genome as described^62^. First, *ttTi5605; unc-119(ed3)* animals derived from the EG6699 strain^61^, which feature uncoordinated (Unc) locomotion, were injected with pIA109 or pIA110 in addition to pCFJ601, pMA122, pGH8, pCFJ90, and pCFJ104. Then NGM plates with non-Unc animals were heat-shocked at 42 °C for 2 h. Non-Unc animals without mCherry fluorescence were selected and subjected to genotyping. PCR was performed to confirm insertion of a full-length transgene.

### Imaging analyses

Calcium imaging was performed as described elsewhere^8,65^. A single adult animal that expressed the genetically encoded calcium indicators R-CaMP2^66^ in AFD and GCaMP3^67^ in AIY or an animal that expressed GCaMP3 and tagRFP in AFD was placed on a 10% agar pad on a cover slip with 0.1 µm polystyrene beads (Polysciences, Warrington, PA, USA) and covered with another cover slip for immobilization^68^. The immobilized animals were placed on a Peltier-based temperature controller (Tokai Hit, Fujinomiya, Japan) on a BX61WI microscope (Olympus, Tokyo, Japan). Red and green fluorescence was separated by the Dual-View optics system (Molecular Devices, Sunnyvale, CA, USA), and images were captured by an ImageEM EM-CCD camera (C9100-13, Hamamatsu Photonics, Japan) at a frame rate of 1 Hz. Excitation pulses were generated by a SPECTRA light engine (Lumencor, Beaverton, OR, USA). Fluorescence intensities were measured using the MetaMorph imaging system (Molecular Devices).

Expression of SLO-2::mCherry was observed with a BX53 upright microscope (Olympus).

### Electrophysiology

HEK293T cells were transfected with plasmids encoding yellow fluorescent protein (YFP) and either the wild-type or H159Y mutant form of SLO-2b with Lipofectamine2000 (Invitrogen). The macroscopic current in HEK293T cells expressing YFP in a whole-cell patch clamp configuration was recorded using the Axopatch 200B amplifiers, Digidata 1322 A, and pClamp 9 software (Axon Instruments, Foster City, CA, USA) at room temperature, as previously described^69^. The bath solution contained (in mM) 140 NaCl, 4 KCl, 10 HEPES, 1 CaCl_2_, and 0.3 MgCl_2_ (pH = 7.4). Pipette resistances ranged from 1 to 3 MΩ when the glass pipette was filled with the pipette solution, which contained (in mM) 130 KCl, 10 NaCl, 10 HEPES, 5 K_2_ATP, 4 MgCl_2_, 3 EGTA, 0.3 GTP, and various concentration of CaCl_2_ (pH = 7.3). To achieve 0, 0.2, 0.6, 2, 6, and 20 µM of free [Ca^2+^]_i_, 1, 1.96, 2.55, 2.86, 2.99, and 3.10 mM of CaCl_2_, respectively, were added to the pipette solution, according to the Ca-Mg-ATP-EGTA Calculator v1.0 using constants from the NIST database #46 v8 (http://maxchelator.stanford.edu/CaMgATPEGTA-NIST.htm). After rupture of the sealed membrane, cells were held at −60 mV for at least 3 min to dialyze the pipette solution into the cell, and then depolarizing step pulses (10 mV increment for 200 ms) were applied. Immediately after application of the step pulse, cells were held at 0 mV (50 ms) for tail current analysis. The current amplitudes were measured at the end of the step pulses and normalized by the membrane capacitance of each cell. Then the current densities were averaged and plotted as a function of the membrane potentials. To analyze the voltage dependence of the channels, the tail current amplitudes at the beginning of 0 mV were measured and normalized by the maximal amplitude at the most depolarized potential (*G*/*G*_max_). *G*/*G*_max_ values were plotted against the membrane potential and fitted to a Boltzmann function to estimate the membrane potential of half-maximal activation (*V*_1/2_) in each cell. The activation kinetics of the channels was analyzed by fitting the traces of outward current to double exponential functions. Several traces that were not fitted to the double exponential functions were eliminated from the kinetic analyses.

### Quantification and statistical analysis

The error bars in histograms and line charts indicate the standard error of mean (SEM). In the boxplots, the bottom and top of boxes represent the first and third quartiles, and the band inside the box represents the median. The ends of the lower and upper whiskers represent the lowest datum still within the 1.5 interquartile range (IQR), which is equal to the difference between the third and first quartiles, of the lower quartile, and the highest datum still within the 1.5 IQR of the upper quartile, respectively. For multiple-comparison tests, one-way analyses of variance were performed, followed by Tukey–Kramer or Dunnett tests, as indicated in each figure legend. The Welch two-sample *t* test was used to compare two values.

## Electronic supplementary material

Supplementary Information

## Data Availability

The datasets generated during the current study, including source data for the figures in the paper, are available in the figshare repository (10.6084/m9.figshare.c.4168931)^70^
