# Supplementary material for: SLO potassium channels antagonize premature decision making in C. elegans
Source: Commun Biol. 2018 Aug 24;1:123. doi: 10.1038/s42003-018-0124-5 (PMC6123717; doi:10.1038/s42003-018-0124-5)
Supplement: Supplementary file 1 — Supplementary Information [file 42003_2018_124_MOESM1_ESM.pdf]

Supplementary Figure 1 (related to Figs. 1 and 3)

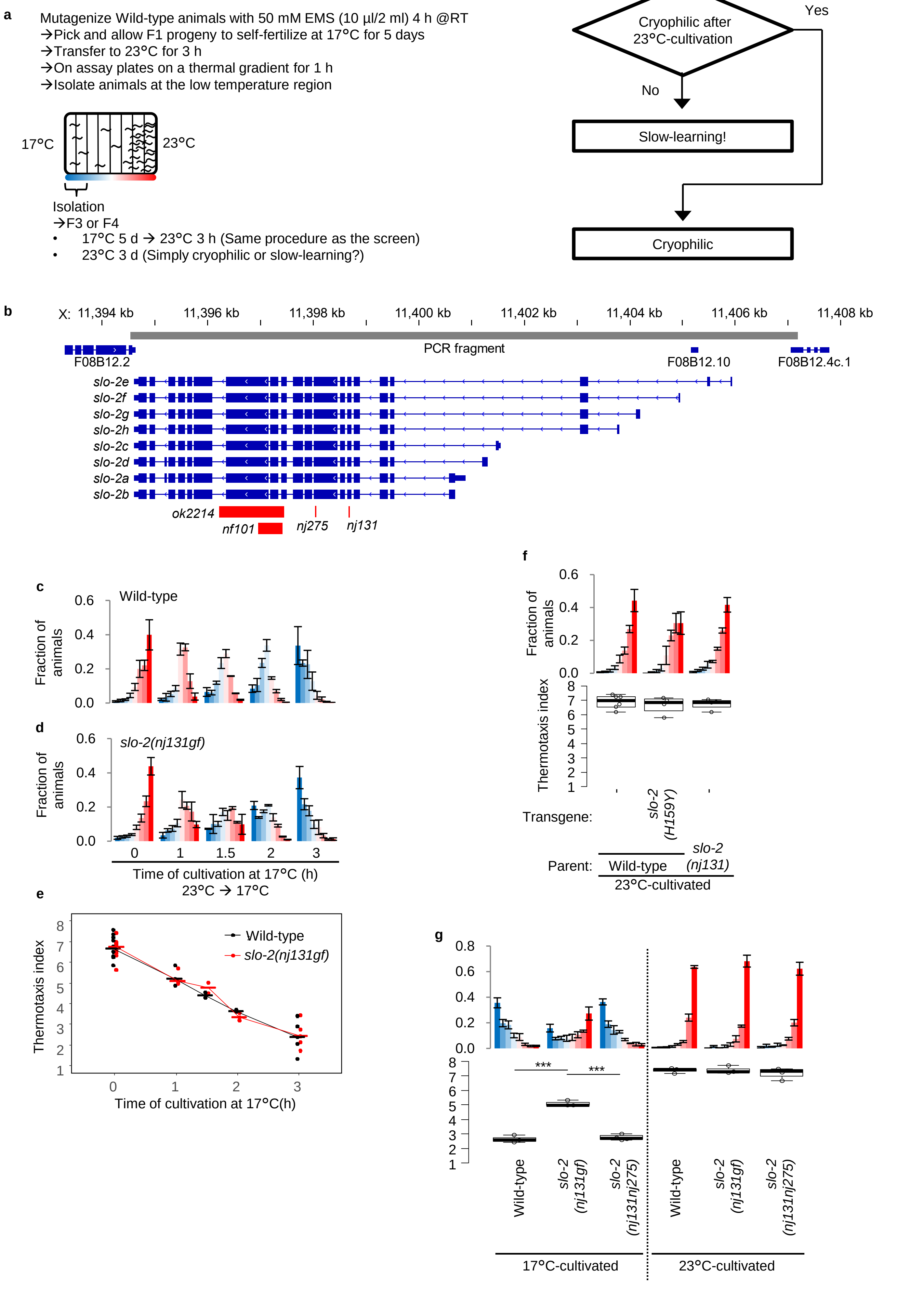

Supplementary Figure 1 (related to Figs. 1 and 3).

a. A schematic drawing of the forward genetic screen for slow-learners is shown.

b. A schematic drawing of the *slo-2* gene locus on the chromosome X is shown.

Using SNP mapping, the *nj131* mutation was mapped between 11.1 Mbp and 12.4 Mbp on linkage group (LG) X. Whole-genome sequencing revealed a single missense mutation within this genomic region, changing the histidine residue 159 of the *slo-2* gene product to tyrosine (H159Y). The genomic locations of the PCR fragment used in Fig. 1k, deletion sites in *ok2214*, *nf101,* and *nj275* alleles, and the site of the *nj131* missense mutation are indicated.

c-e. *slo-2(nj131gf)* does not affect thermotaxis after a downshift in temperature.

Wild type (c) and *slo-2(nj131gf)* (d) animals were cultivated at 23°C for 3 days and then at 17°C for the time indicated. The animals were then subjected to thermotaxis assay. The thermotaxis indices at each time point in c and d are plotted against time after the cultivation temperature was changed to 23°C (e). Horizontal bars indicate medians. n = 10, 3, 2, 3, 5 for each time point.

f. Wild-type, *slo-2(nj131gf)*, and wild-type animals injected with genomic PCR fragments covering the *slo-2* gene locus derived from *nj131* mutants were cultivated constantly at 23°C for 3 days and then subjected to thermotaxis assay. n = 6, 4, 4.

g. Wild-type, *slo-2(nj131gf)*, and *slo-2(nj131nj275)* animals were cultivated constantly at 17°C for 5 days or at 23°C for 3 days and then subjected to thermotaxis assay. n = 3, 3, 4 for each strain. ***p < 0.001 (Tukey-Kramer test).

The error bars in histograms and line charts represent the SEM.

Supplementary Figure 2 (related to Figs. 3 and 8)

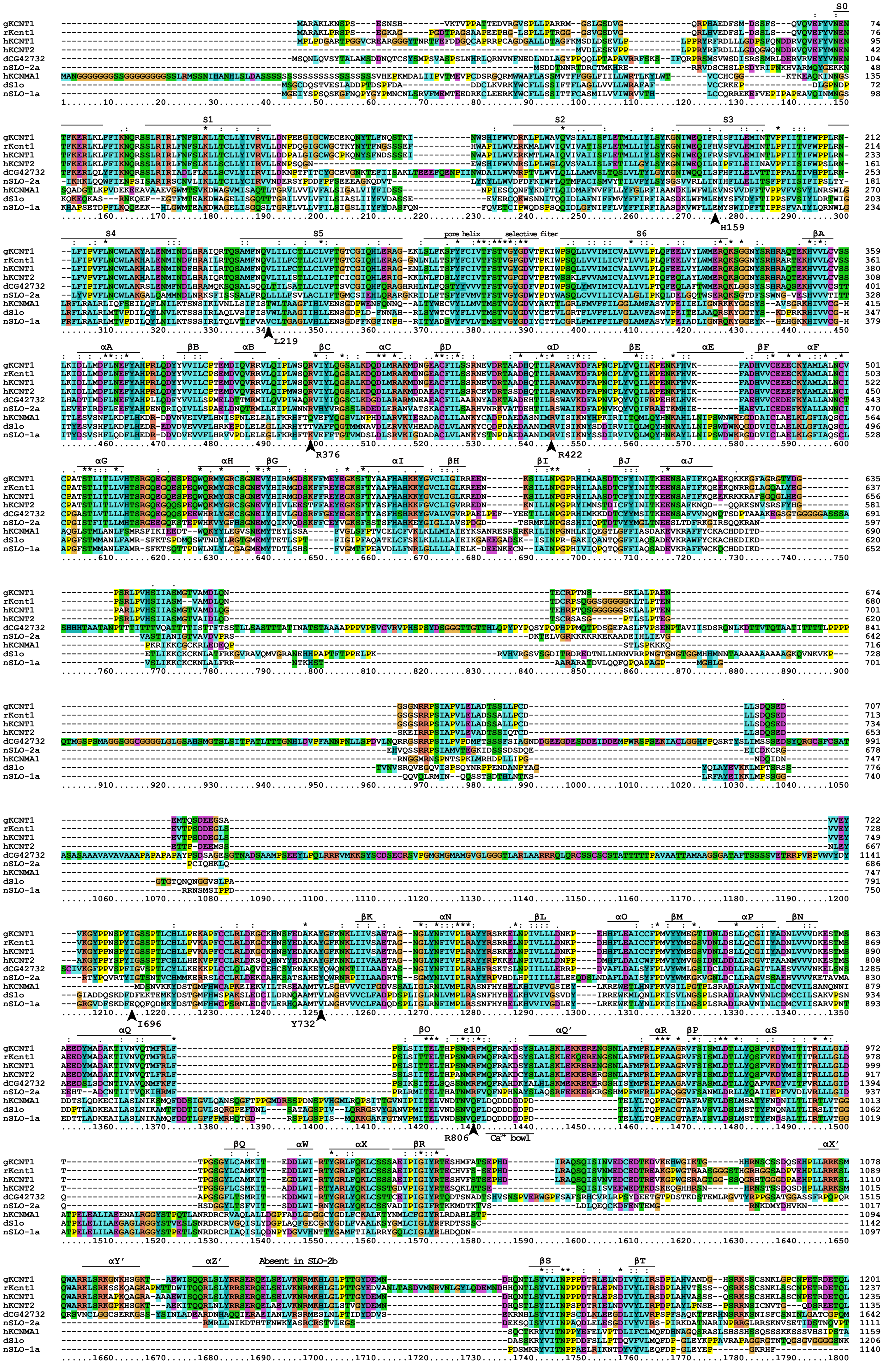

Supplementary Figure 2 (related to Figs. 3 and 8). SLO-2 and SLO-1 amino acid sequences are aligned.

Amino acid sequences of chicken KCNT1 (gKCNT1, NP_989893.1), rat Kcnt1 (rKCNT1, NP_068625.1), human KCNT1 (hKCNT1, NP_001258932.1), human KCNT2 (hKCNT2, NP_940905.2), fruit fly SLO2 (dCG42732, NP_001097259.2), *C. elegans* SLO-2 isoform a (nSLO-2a, NP_001024527.1), human KCNMA1 (hKCNMA1, NP_001014797.1), fruit fly slo (dslo, NP_001163712.1), and *C. elegans* SLO-1 isoform a (nSLO-1a, NP_001024259.1) were aligned with ClustalX2. The sequences of the RCK2 domain were then manually aligned according to the structural information [1].

Helices S0 to S6 and the selective filter in the transmembrane domain; αA to αJ and βA to βJ in the RCA1 domain; αN to αZ' and βK to βT in the RCA2 domain; "Ca^{2+} bowl" in KCNMA1; and amino acid residues present in SLO-2a but absent in SLO-2b are indicated. H159, which is mutated by *slo-2(nj131gf)*, and epilepsy-related mutation sites (shown in Fig. 8) are indicated by arrowheads.

Supplementary Figure 3 (related to Figs. 3)

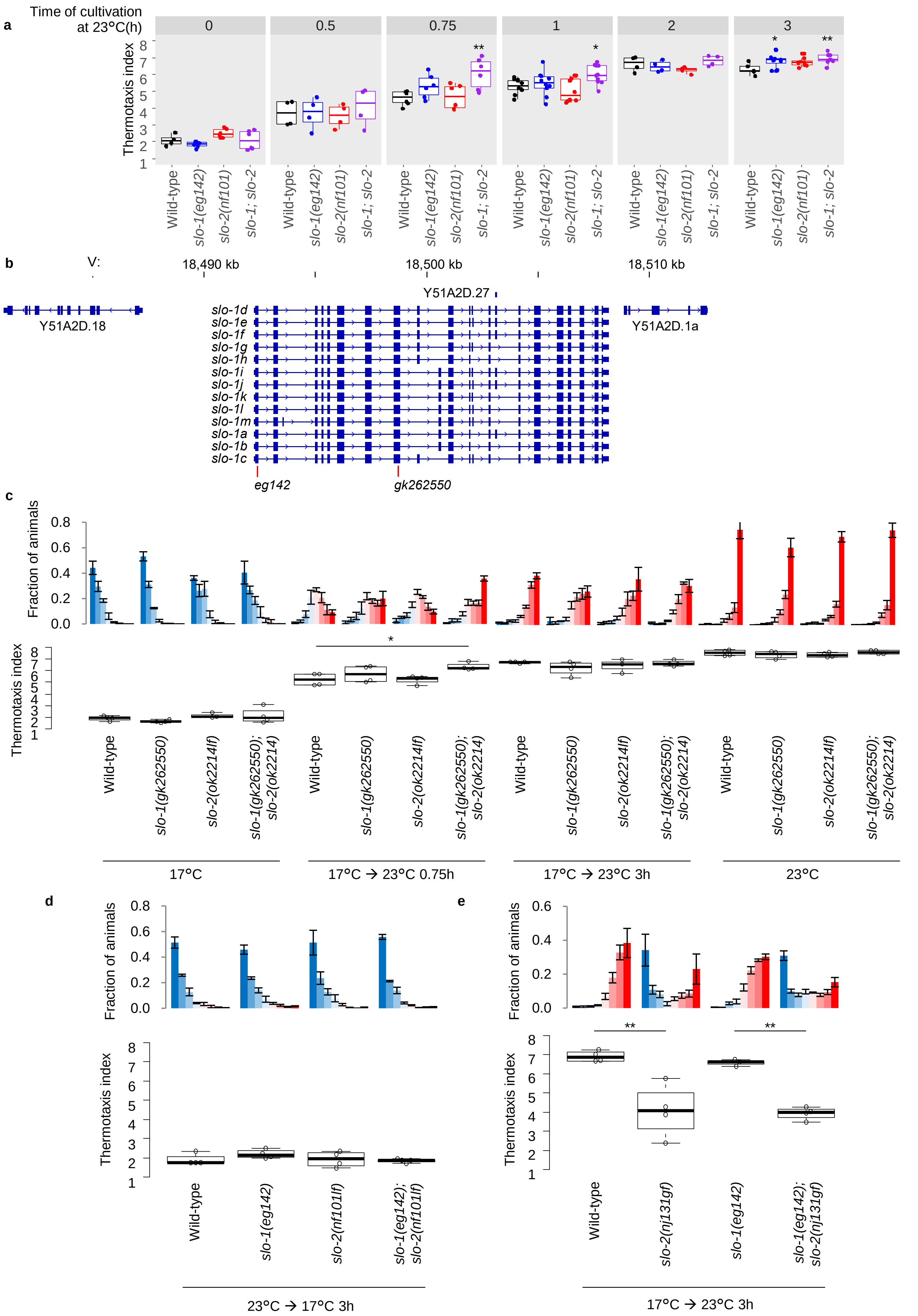

Supplementary Figure 3 (related to Fig. 3).

a. Individual data points in Fig. 3 are plotted

b. A schematic drawing of the *slo-1* gene locus on the chromosome V is shown. The sites of *eg142* and *gk262550* nonsense mutations are indicated.

c. The thermotaxis behavior of *slo-1; slo-2* double lf mutants is quick to change.

Wild-type, *slo-1(gk262550)*, *slo-2(ok2214lf)*, and *slo-1(gk262550); slo-2(ok2214lf)* animals were cultivated constantly at 17°C for 5 days, at 17°C for 5 days and then at 23°C for 45 min or 3 hours or constantly at 23°C for 3 days. Animals were then subjected to thermotaxis assay. n = 3 for *slo-2(ok2214)* cultivated constantly at 17°C or at 17°C and then at 23°C for 3 hours, and n = 4 for others. *$p < 0.05$ (Dunnett test).

d. Wild-type, *slo-1(eg142)*, *slo-2(nf101lf)*, and *slo-1(eg142); slo-2(nf101lf)* animals were cultivated at 23°C for 3 days and then at 17°C for 3 hours. Animals were then subjected to thermotaxis assay. n = 4.

e. Wild-type, *slo-1(eg142)*, *slo-2(nj131gf)*, and *slo-1(eg142); slo-2(nj131gf)* animals were cultivated at 17°C for 5 days and then at 23°C for 3 hours. Animals were then subjected to thermotaxis assay. n = 4, 4, 3, 4. **$p < 0.01$ (Tukey-Kramer test).

The error bars in histograms represent SEM.

Supplementary Figure 4 (related to Fig. 4)

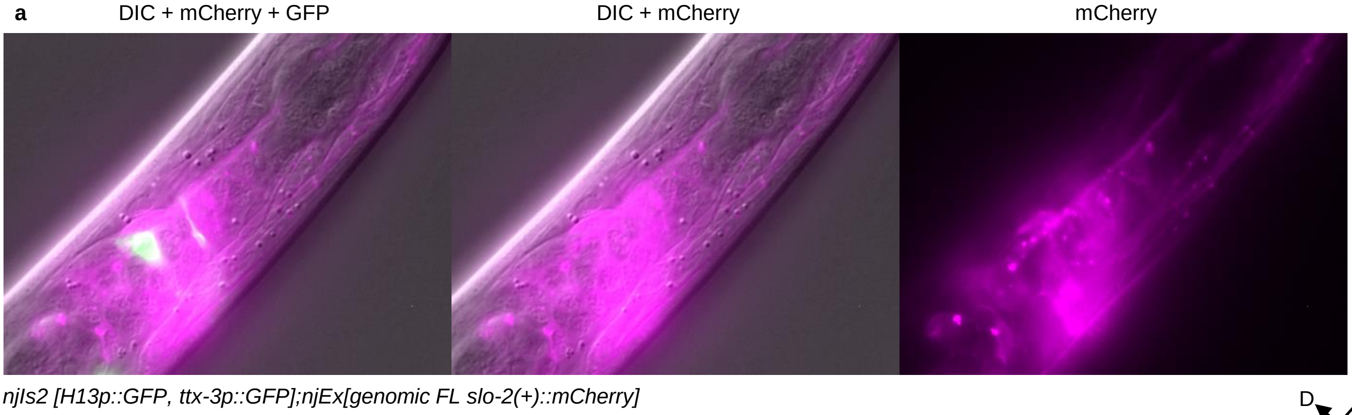

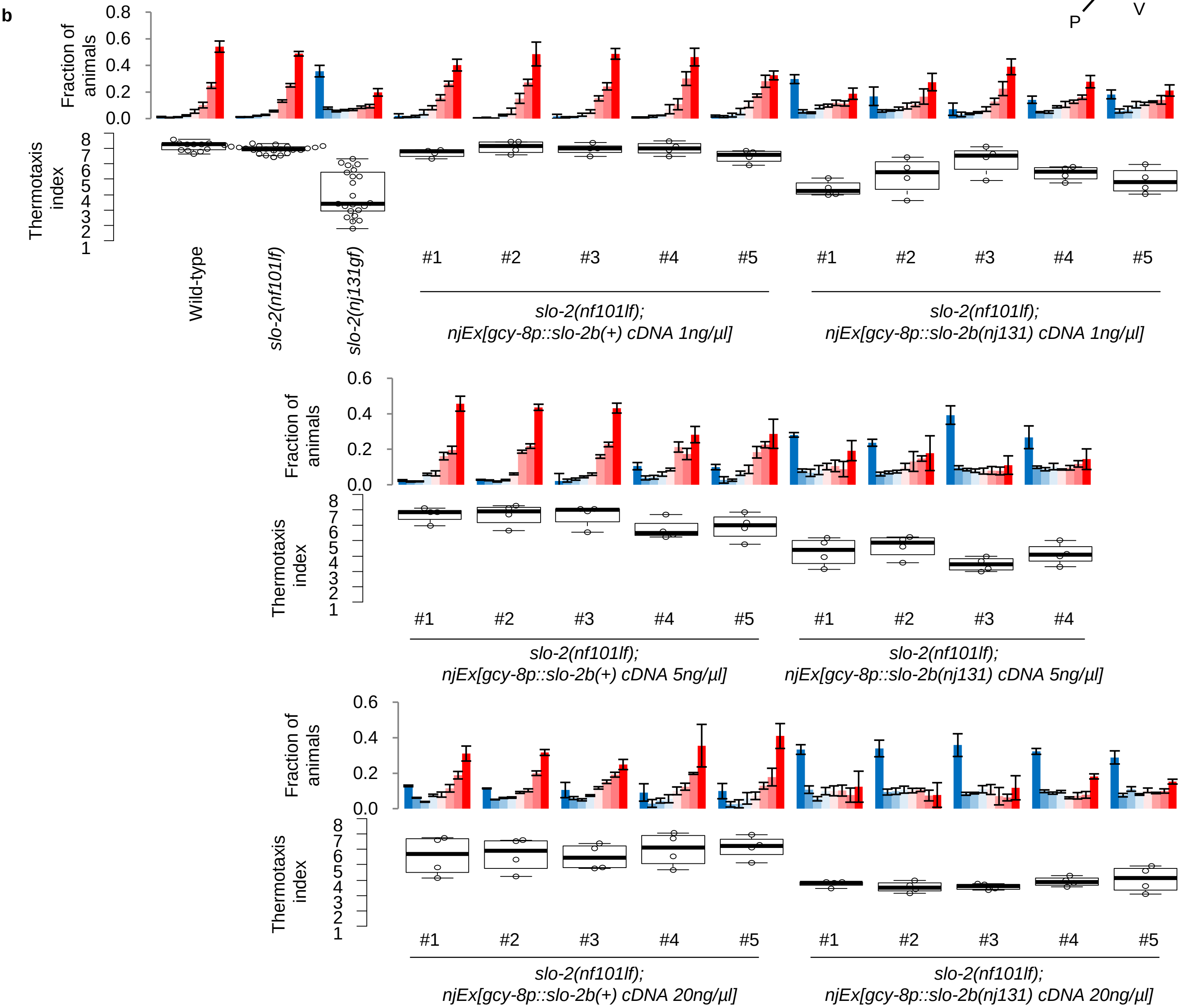

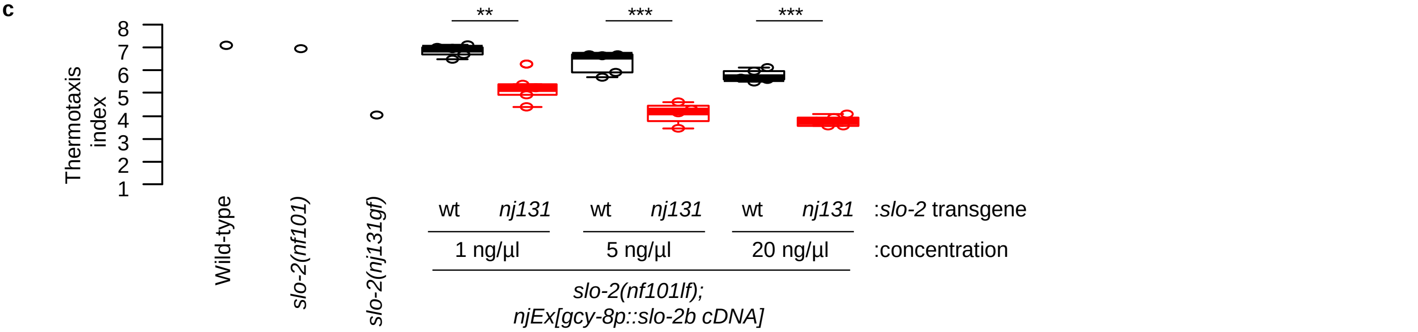

Supplementary Figure 4 (related to Fig. 4).

a. Expression of a full-length genomic *slo-2::mCherry* fusion gene.

Animals expressing green fluorescent protein (GFP) in AFD and AIY neurons were injected with *slo-2::mCherry*. Animals were then subjected to microscopic analysis with Olympus BX53. Merged differential interference contrast (DIC), GFP, and mCherry image (left). Merged DIC and mCherry image (middle) and mCherry image (right) of the same animal in the left image. *slo-2::mCherry* expression was observed in AFD. AIY soma was out of the field of view.

b. The H159Y mutant form of SLO-2 more potently decelerate behavior transition than the wild-type form.

slo-2(nf101lf) animals were injected with plasmids encoding either wild type or H159Y mutant form of SLO-2 downstream of *gcy-8* promoter at concentrations of 1, 5 or 20 ng/μl. Animals were cultivated at 17°C for 5 days and then at 23°C for 3 hours and subjected to thermotaxis assay. # indicates independent transgenic lines. n = 11 for wild type, n = 22 for *slo-2(nf101)* and *slo-2(nj131gf),* and n = 4 for the rest.

c. The thermotaxis indices of each strain in B are plotted against the concentrations of the injected plasmids. $^{**}p < 0.01$, $^{***}p < 0.001$ (Welch two-sample t-test).

Two of the three strains that were injected with a genomic *slo-2* locus derived from *nj131* animals with a wild-type background (Fig. 1g) showed uncoordinated (Unc) locomotion, while all of three strains expressing wild-type SLO-2 did not. These results imply that the mutant form might possess higher or aberrant activity compared to the wild-type form and are consistent with electrophysiological (Fig. 2) and behavioral (Supplementary Fig. 4b and c) experiments, which revealed the higher activity of the mutant channel.

Supplementary Figure 5 (related to Fig. 5)

a

Mutagenize *slo-2(nj131gf)* animals with 50 mM EMS (10 µl/2 ml) 4 h @RT
→Pick and allow F1 progeny to self-fertilize at 17°C for 5 days
→Transfer to 23 °C for 3 h
→On assay plates on a thermal gradient for 1 h
→Isolate animals at the **high** temperature region

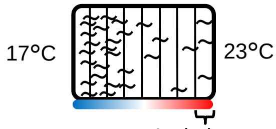

→F3 or F4
- 17°C 5 d → 23°C 3 h (Same procedure as the screen)
- 17°C 5 d (Simply thermophilic or suppressor?)

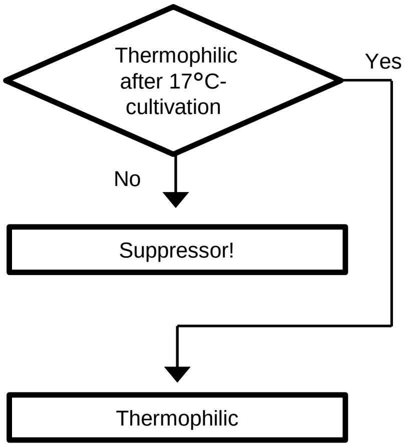

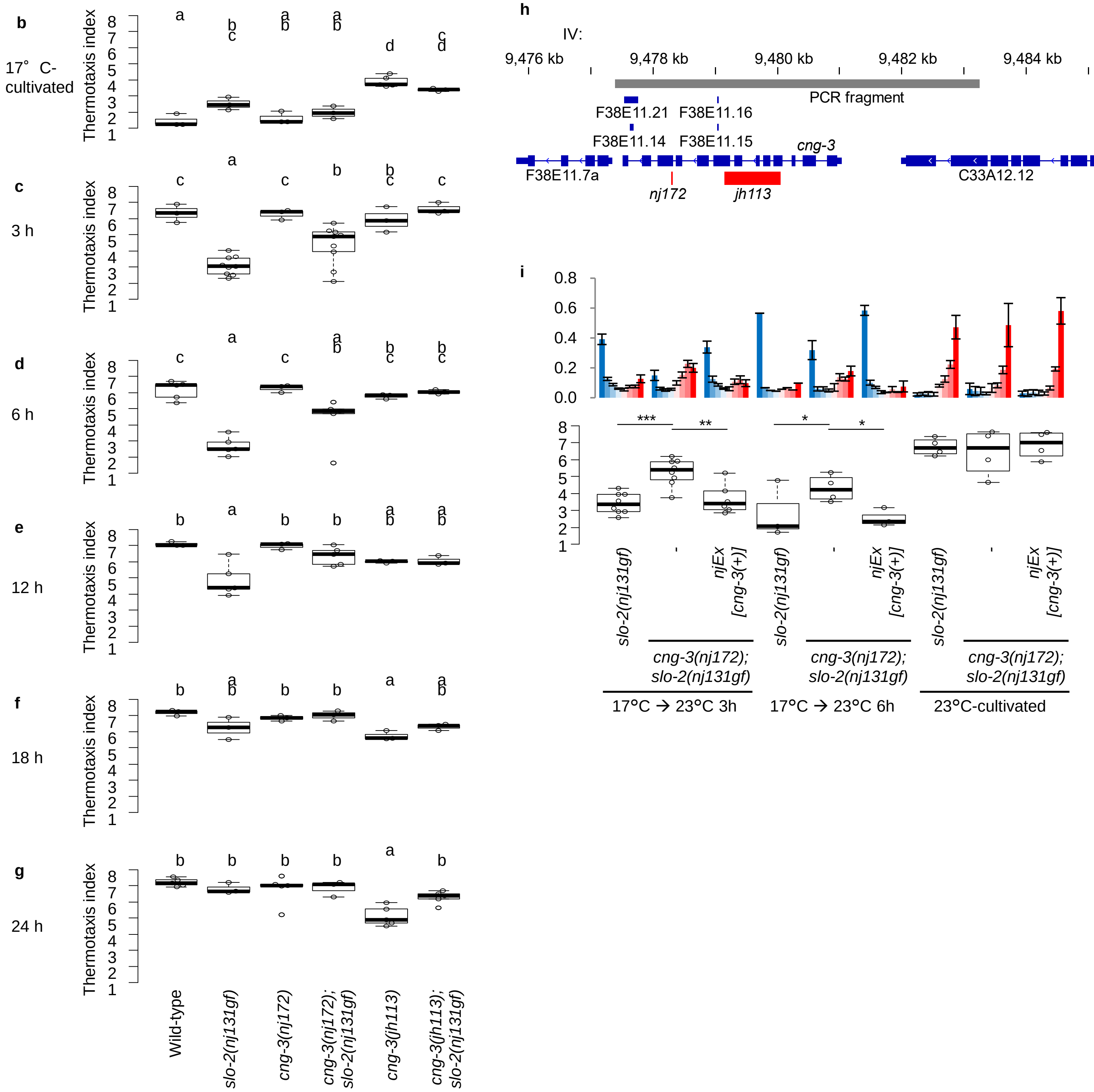

Supplementary Figure 5 (related to Fig. 5).

a. A schematic drawing of the screen for suppressors of *slo-2(nj131gf)* is shown.

b-g. The thermotaxis indices at each time point in the experiment in Fig. 5a–g are shown. Means of indices of strains marked with distinct alphabets differ significantly ($p < 0.05$) according to the Tukey-Kramer test.

h. A schematic drawing of the *cng-3* gene locus is shown.

Using SNP mapping, the *nj172* mutation was mapped between 8.7 Mbp and 9.8 Mbp on LG IV. Whole-genome sequencing revealed a missense mutation within this genomic region, which changes the methionine residue 467 of the *cng-3* gene product to isoleucine (M467I). The genomic locations of the PCR fragment used in Fig. 5i, the *jh113* deletion site, and the site of the *nj172* missense mutation are indicated.

i. *slo-2(nj131gf), cng-3(nj172); slo-2(nj131gf)*, and *cng-3(nj172); slo-2(nj131gf)* animals that were injected with genomic PCR fragments covering the *cng-3* locus were cultivated first at 17°C then at 23°C for the time indicated or constantly at 23°C, and then subjected to thermotaxis assay. n = 8, 8, 6, 3, 4, 3, 4, 4, 4. *$p < 0.05$, **$p < 0.01$, ***$p < 0.001$ (Tukey-Kramer test).

Supplementary Figure 6 (related to Fig. 5)

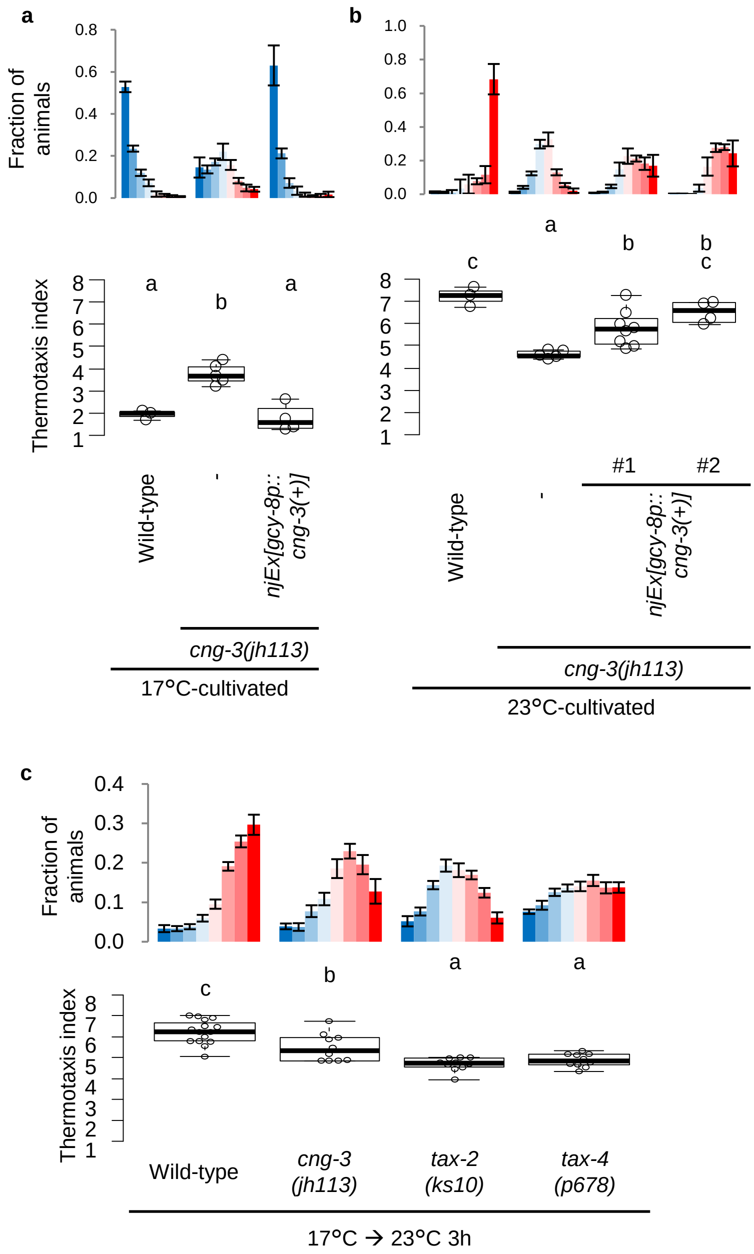

Supplementary Figure 6 (related to Fig. 5).

a, b. Wild-type and *cng-3(jh113)* animals and *cng-3(jh113)* animals expressing *cng-3* under control of the *gyc-8* promoter were cultivated at 17°C for 5 days (a), or at 23°C for 3 days (b), and then subjected to thermotaxis assay. n = 3, 5, 4 for a, and n = 3, 5, 8, 4 for b. Means of indices of strains marked with distinct alphabets differ significantly ($p < 0.05$) according to the Tukey-Kramer test.

c. Wild-type, *cng-3(jh113), tax-2(ks10)*, and *tax-4(p678)* animals were cultivated at 17°C for 5 days and then at 23°C for 3 hours. Animals were then subjected to thermotaxis assay. n = 13, 10, 10, 10. Means of indices of strains marked with distinct alphabets differ significantly ($p < 0.05$) according to the Tukey-Kramer test.

Supplementary Figure 7 (related to Fig. 8)

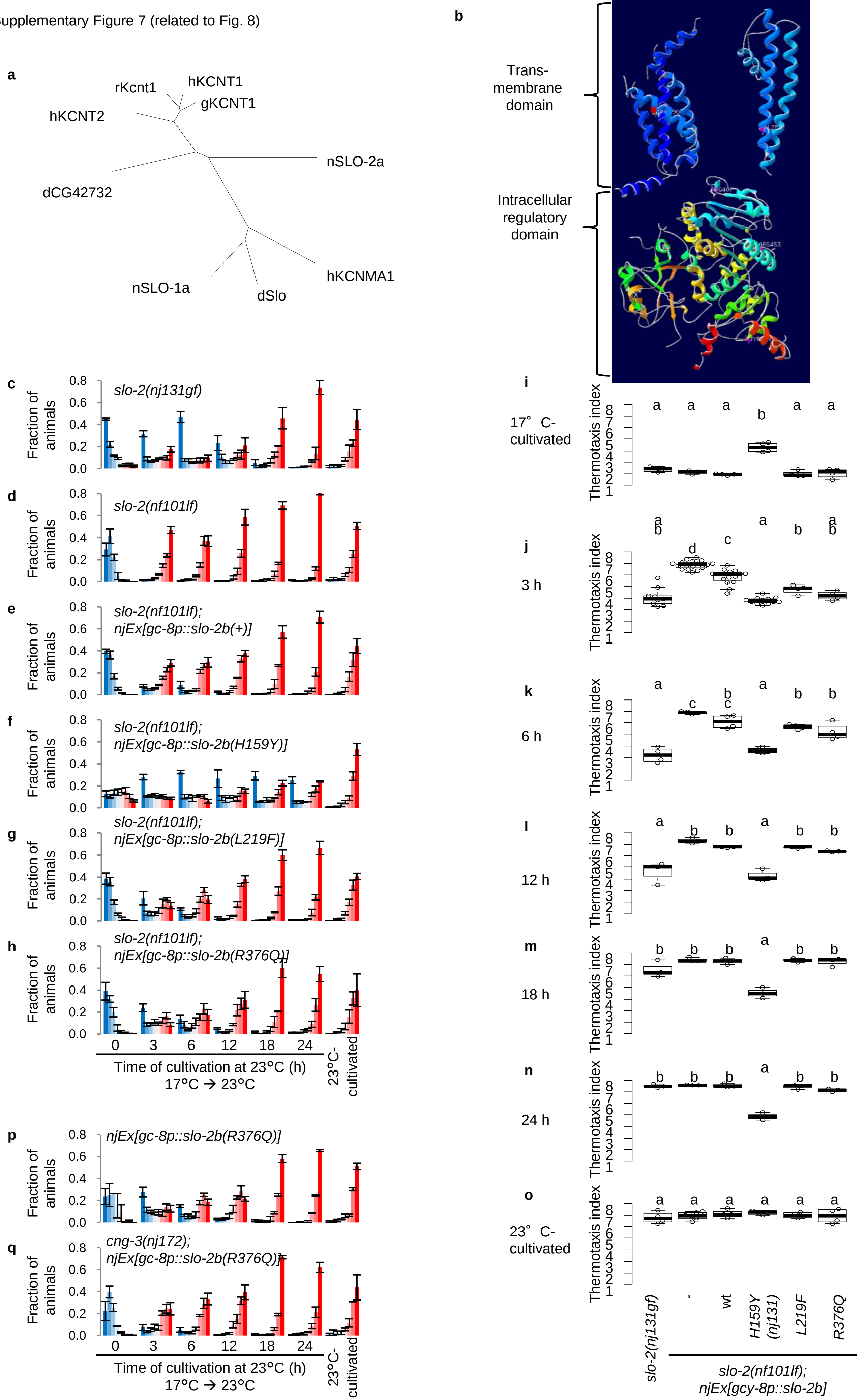

Supplementary Figure 7 (related to Fig. 8).

a. The phylogenic tree for SLO-1 and SLO-2 homologs was generated by ClustalX2 and visualized with TreeView.

b. The structure of chicken KCNT1 [1] is visualized with a Swiss PDB Viewer. Amino acid residues corresponding to H159 in *C. elegans* SLO-2 and four epilepsy-related mutations introduced in Fig. 8, which are located on the solved structure, are indicated by red and pink, respectively.

c-h. The distribution of animals on thermal gradients in the experiment shown in Fig. 8b is plotted on histograms. *slo-2(nj131gf)* mutants (c, n = 5, 9, 4, 3, 3, 3, 4), *slo-2(nf101)* mutants (d, n = 3, 17, 4, 3, 3, 3, 6), *slo-2(nf101lf)* animals that expressed wild-type (e, n = 4, 16, 4, 3, 3, 3, 4), H159Y (f, n = 4, 12, 4, 3, 3, 3, 4), L219F (g, n = 4, 4, 4, 3, 3, 3, 4), or R376Q (h, n = 4, 4, 4, 3, 3, 3, 4) mutant forms of SLO-2b in AFD were cultivated at 17°C for 5 days and then at 23°C for the time indicated or constantly at 23°C for 3 days. Animals were then subjected to thermotaxis assay. The data at 3 hours are identical to those in Fig. 8a.

i-o. The thermotaxis indices at each time point in a–f are plotted. The means of indices of strains marked with distinct alphabets differ significantly ($p < 0.05$) according to the Tukey-Kramer test.

p, q. The distribution of animals on thermal gradients in the experiment shown in Fig. 8c is plotted in histograms. Animals that expressed SLO-2b(R376Q) in AFD with either a wild-type (p) or *cng-3(nj172)* (q) background were cultivated at 17°C for 5 days and then at 23°C for the time indicated, or constantly at 23°C for 3 days. Animals were then subjected to thermotaxis assay. n = 2, 6, 3, 5, 3, 3, 2 for each time point.

Supplementary Figure 8 (related to Fig. 8)

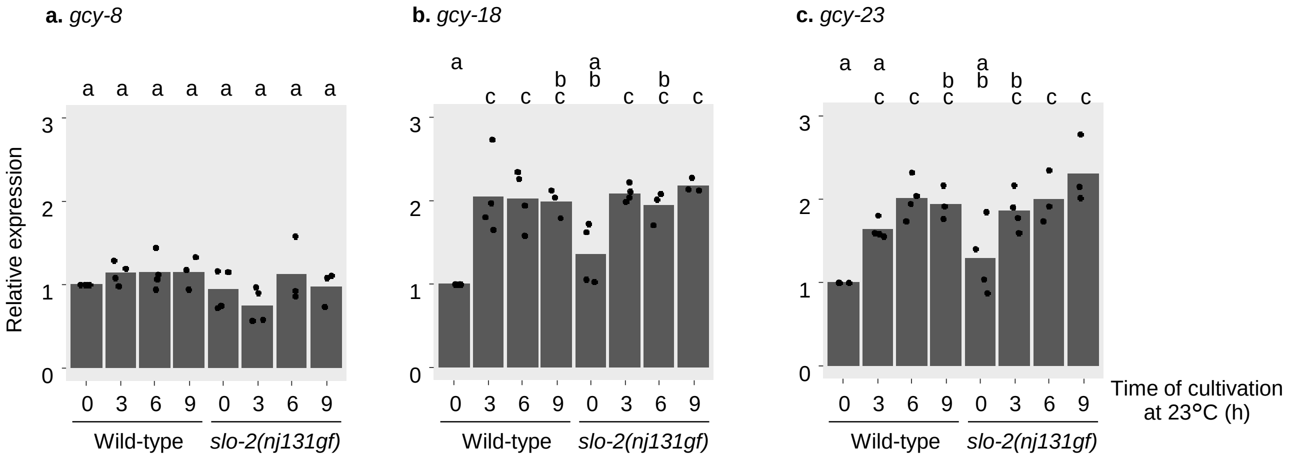

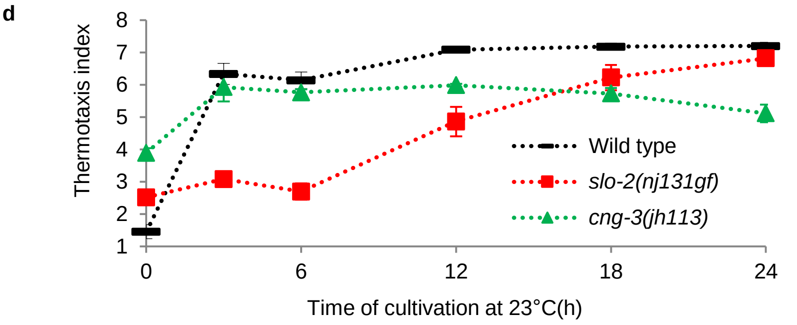

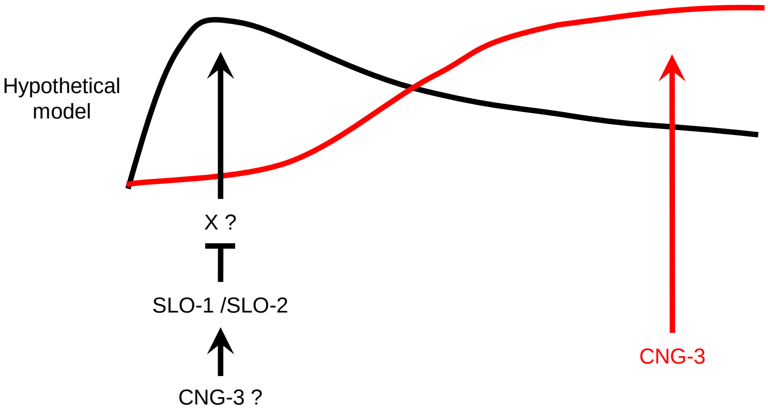

Supplementary Figure 8 (related to Fig. 8).

a-c. Induction of *gcy* expression by temperature upshift was not affected by *slo-2(nj131gf)* mutation.

Previous studies suggested that the amount of cGMP might determine the onset temperature of AFD [2,3]. Since the expression levels of AFD-specific guanylyl cyclases (GCs), GCY-8, GCY-18 and GCY-23, were shown to increase after an upshift in cultivation temperature [4], we examined whether the increase of these GCs was hampered in *slo-2(nj131gf)* animals.

Wild-type or *slo-2(nj131gf)* animals were cultivated at 17°C for 5 days and then at 23°C for the time indicated. Total RNA was prepared, and a cDNA library was constructed using reverse transcription. The amounts of *gcy-8* (a), *gcy-18* (b), and *gcy-23* (c) cDNA were quantified using real-time PCR analysis. The expression levels of each gene were normalized by the geometric means of the expression levels of internal controls in each sample. Next, the expression levels were normalized by the expression levels in wild-type animals cultivated constantly at 17°C in each experiment. The normalized expression levels of each experiment are shown by dots and the means are shown by bars. n = 3 or 4. Values marked with distinct alphabets differ significantly ($p < 0.05$) according to the Tukey-Kramer test.

The expression levels of *gcy-18* and *gcy-23* increased after an upshift of cultivation temperature from 17°C to 23°C in both wild-type and *slo-2(nj131gf)* animals (b and c). Neither wild-type nor *slo-2(nj131gf)* animals showed a significant increase in the level of *gcy*-8 mRNA (a). These results suggest that SLO-2 slows down AFD adaptation and temperature preference transition in parallel to or downstream of regulation of GC expression.

d. Thermotaxis transition might involve both “fast and transient” and “slow and persistent” components.

The thermotaxis indices are identical to those in Fig. 5g (upper).

Supplementary Table 1. Primers used for qPCR (related to Supplementary Figure 8).

**Gene**	**Forward**	**Reverse**
act-1	CGCCAACACTGTTCTTTCCG	TCATGGTTGATGGGGCAAGAG
gpd-1	AGGGAATCCTCGCCTACACT	TTGTCGTACCAAGAGACGAGC
lmn-1	GAAGCTCGCTCCACATGCTA	TCCAATTGGCCACTGTTGCT
gcy-8	AGAAGCCAACCAACGAGCAG	CCGACAATGTCACTGAAAAGCAC
gcy-18	CGAGGACAGAGATGCAGTTACC	GGAAAATTCGCCAAGGCATC
gcy-23	CAATGTGCGAGCCGACAAAC	AGCCCACGATGTCACTGAAC
slo-2	TGGAGTGGAATCTCATGTTGTTGTG	TCTCTGGTTTTCGGGGTGGG
cng-3	CAGATGCTTACTTTGTTGGCTGTG	ATGGGTTCTGTTATCCGTTCTCC

Supplementary Table 2. Strain list.

Description	Source	Identifier
C. elegans: wild isolate	CGC	WormBase: N2 RRID:WB-STRAIN:N2_(ancestral)
C. elegans: wild isolate	CGC	WormBase: CB4858
slo-2(nj131gf)	This paper	IK1671
cng-3(nj172)	This paper	IK2321
cng-3(nj172); slo-2(nj131gf)	This paper	IK2320
cng-3(jh113)	This paper; KJ462 [5]; CGC	IK2303
cng-3(jh113); slo-2(nj131gf)	This paper	IK2366
njEx675[slo-2(+) genomic PCR fragment 5 ng/ul, ges-1p::NLS::GFP]	This paper	IK1829
njEx678[slo-2(nj131) genomic PCR fragment 5 ng/ul, ges-1p::NLS::GFP]	This paper	IK1832
slo-2(nj131gf); njEx680[slo-2(+) genomic PCR fragment 5 ng/ul, ges-1p::NLS::GFP]	This paper	IK1834
slo-2(nj131nj275)	This paper	IK2801
slo-2(nj131gf) (CB4858 background)	This paper	IK2092
cng-3(nj172); slo-2(nj131gf); njEx882[cng-3(+) genomic PCR fragment 5 ng/ul, ges-1p::NLS::GFP]	This paper	IK2311
slo-1(eg142)	This paper; BZ142 [6]; CGC	IK2607
slo-2(nf101)	This paper; LY101 [7]; CGC	IK2302
slo-1(eg142); slo-2(nf101)	This paper	IK1910
slo-1(eg142); slo-2(nj131)	This paper	IK3153
ttTi5605; unc-119(ed3); oxEx1578	[8]; CGC	EG6699
njSi7[gyc-8p::slo-1a(+), Cb-unc-119(+)]; unc-119(ed3)	This paper	IK3208
njSi7[gyc-8p::slo-1a(+), Cb-unc-119(+)]; slo-1(eg142); slo-2(nf101)	This paper	IK3209
njSi8[gyc-8p::slo-2b(+), Cb-unc-119(+)]; unc-119(ed3)	This paper	IK3416
njSi8[gyc-8p::slo-2b(+), Cb-unc-119(+)]; slo-1(eg142); slo-2(nf101)	This paper	IK3417
slo-1(gk262550)	This paper; VC20278; CGC	IK3204
slo-2(ok2214)	This paper; VC1819 [9]; CGC	IK3182
slo-1(gk262550); slo-2(ok2214)	This paper	IK3205
njEx1102[snb-1p::slo-2b(nj131) 20ng/ul]	This paper	IK2802
njEx1098[gcy-8p::SI::slo-2b(nj131) 20ng/ul]	This paper	IK2787
njEx1107[ceh-36p::SI::slo-2b(nj131) 20ng/ul]	This paper	IK2815
njEx1109[myo-3p::SI::slo-2b(nj131) 20ng/ul]	This paper	IK2817
njEx1111[ttx-3p::SI::slo-2b(nj131) 20ng/ul, glr-3p::SI::slo-2b(nj131)20ng/ul, lin-11p::SI::slo-2b(nj131) 20ng/ul]	This paper	IK2819
cng-3(nj172); slo-2(nj131gf); njEx1143[gcy-8p::SI::cng-3 20ng/ul]	This paper	IK2850
cng-3(nj172); slo-2(nj131gf); njEx1144[gcy-8p::SI::cng-3 20ng/ul]	This paper	IK2851
cng-3(nj172); slo-2(nj131gf); njEx1146[snb-1p::SI::cng-3 20ng/ul]	This paper	IK2853
cng-3(nj172); slo-2(nj131gf); njEx1147[ceh-36p::SI::cng-3 20ng/ul]	This paper	IK2854
cng-3(nj172); slo-2(nj131gf); njEx1148[ceh-36p::SI::cng-3 20ng/ul]	This paper	IK2855
cng-3(jh113); slo-2(nj131gf); njEx1143[gcy-8p::SI::cng-3 20ng/ul]	This paper	IK3083
cng-3(jh113); slo-2(nj131gf); njEx1144[gcy-8p::SI::cng-3 20ng/ul]	This paper	
njEx1062[gcy-8p::R-CaMP2, AIYp::GCaMP3, ges-1p::nls-	This paper	IK2628

TagRFP]		
slo-2(nj131gf); njEx1062[gcy-8p::R-CaMP2, AIYp::GCaMP3, ges-1p::nls-TagRFP]	This paper	IK2655
cng-3(jh113); njEx1062[gcy-8p::R-CaMP2, AIYp::GCaMP3, ges-1p::nls-TagRFP]	This paper	IK2806
cng-3(jh113); slo-2(nj131gf); njEx1062[gcy-8p::R-CaMP2, AIYp::GCaMP3, ges-1p::nls-TagRFP]	This paper	IK2807
njEx1099[gcy-8p::SI::slo-2b(nj131) 20ng/ul]; njEx1062[gcy-8p::R-CaMP2, AIYp::GCaMP3, ges-1p::nls-TagRFP]	This paper	IK2882
cng-3(jh113); slo-2(nj131gf); njEx1143[gcy-8p::cng-3(+)]; njEx1062[gcy-8p::R-CaMP2, AIYp::GCaMP3, ges-1p::nls-TagRFP]	This paper	IK3081
njIs24[gcy-8p::GCaMP3, gcy-8p::TagRFP]	[10]	IK0961
njIs24[gcy-8p::GCaMP3, gcy-8p::TagRFP]; slo-2(nj131gf)	This paper	IK1738
njIs24[gcy-8p::GCaMP3, gcy-8p::TagRFP]; slo-1(eg142)	This paper	IK2674
njIs24[gcy-8p::GCaMP3, gcy-8p::TagRFP]; slo-2(nf101)	This paper	IK2616
njIs24[gcy-8p::GCaMP3, gcy-8p::TagRFP]; slo-1(eg142); slo-2 (nf101)	This paper	IK2675
njIs24[gcy-8p::GCaMP3, gcy-8p::TagRFP]; cng-3(jh113)	This paper	IK3207
slo-2(nf101); njEx1172[gcy-8p::SI::slo-2b(L219F) 20ng/ul]	This paper	IK2923
slo-2(nf101); njEx1174[gcy-8p::SI::slo-2b(R376Q) 20ng/ul]	This paper	IK2925
slo-2(nf101); njEx1176[gcy-8p::SI::slo-2b(I696M) 20ng/ul]	This paper	IK2927
slo-2(nf101); njEx1177[gcy-8p::SI::slo-2b(R866C) 20ng/ul]	This paper	IK2928
slo-2(nf101); njEx1178[gcy-8p::SI::slo-2b(F870I) 20ng/ul]	This paper	IK2929
slo-2(nf101); njEx1114[gcy-8p::SI::slo-2b(+) 20ng/ul]	This paper	IK2821
slo-2(nf101); njEx1115[gcy-8p::SI::slo-2b(+) 20ng/ul]	This paper	IK2822
slo-2(nf101); njEx1116[gcy-8p::SI::slo-2b(+) 20ng/ul]	This paper	IK2823
slo-2(nf101); njEx1117[gcy-8p::SI::slo-2b(+) 20ng/ul]	This paper	IK2824
slo-2(nf101); njEx1118[gcy-8p::SI::slo-2b(+) 20ng/ul]	This paper	IK2825
slo-2(nf101); njEx1119[gcy-8p::SI::slo-2b(+) 5ng/ul]	This paper	IK2826
slo-2(nf101); njEx1120[gcy-8p::SI::slo-2b(+) 5ng/ul]	This paper	IK2827
slo-2(nf101); njEx1121[gcy-8p::SI::slo-2b(+) 5ng/ul]	This paper	IK2828
slo-2(nf101); njEx1122[gcy-8p::SI::slo-2b(+) 5ng/ul]	This paper	IK2829
slo-2(nf101); njEx1123[gcy-8p::SI::slo-2b(+) 5ng/ul]	This paper	IK2830
slo-2(nf101); njEx1124[gcy-8p::SI::slo-2b(+) 1ng/ul]	This paper	IK2831
slo-2(nf101); njEx1125[gcy-8p::SI::slo-2b(+) 1ng/ul]	This paper	IK2832
slo-2(nf101); njEx1126[gcy-8p::SI::slo-2b(+) 1ng/ul]	This paper	IK2833
slo-2(nf101); njEx1127[gcy-8p::SI::slo-2b(+) 1ng/ul]	This paper	IK2834
slo-2(nf101); njEx1128[gcy-8p::SI::slo-2b(+) 1ng/ul]	This paper	IK2835
slo-2(nf101); njEx1129[gcy-8p::SI::slo-2b(nj131) 20ng/ul]	This paper	IK2836
slo-2(nf101); njEx1130[gcy-8p::SI::slo-2b(nj131) 20ng/ul]	This paper	IK2837
slo-2(nf101); njEx1131[gcy-8p::SI::slo-2b(nj131) 20ng/ul]	This paper	IK2838
slo-2(nf101); njEx1132[gcy-8p::SI::slo-2b(nj131) 20ng/ul]	This paper	IK2839
slo-2(nf101); njEx1133[gcy-8p::SI::slo-2b(nj131) 20ng/ul]	This paper	IK2840
slo-2(nf101); njEx1134[gcy-8p::SI::slo-2b(nj131) 5ng/ul]	This paper	IK2841
slo-2(nf101); njEx1135[gcy-8p::SI::slo-2b(nj131) 5ng/ul]	This paper	IK2842
slo-2(nf101); njEx1136[gcy-8p::SI::slo-2b(nj131) 5ng/ul]	This paper	IK2843
slo-2(nf101); njEx1137[gcy-8p::SI::slo-2b(nj131) 5ng/ul]	This paper	IK2844
slo-2(nf101); njEx1138[gcy-8p::SI::slo-2b(nj131) 1ng/ul]	This paper	IK2845
slo-2(nf101); njEx1139[gcy-8p::SI::slo-2b(nj131) 1ng/ul]	This paper	IK2846
slo-2(nf101); njEx1140[gcy-8p::SI::slo-2b(nj131) 1ng/ul]	This paper	IK2847
slo-2(nf101); njEx1141[gcy-8p::SI::slo-2b(nj131) 1ng/ul]	This paper	IK2848
slo-2(nf101); njEx1142[gcy-8p::SI::slo-2b(nj131) 1ng/ul]	This paper	IK2849

Supplementary Table 3. Plasmid list.

Description	Source	Identifier
Peft-3::cas9-SV40_NLS::tbb-2 3'UTR	[11]	Addgene plasmid #46168
PU6::unc-119_sgRNA	[11]	Addgene plasmid #46169
PU6::unc-22_sgRNA	[12]	pSN680
PU6::slo-2_sgRNA #1	This paper	pIA009
PU6::slo-2_sgRNA #4	This paper	pIA054
rol-6(su1006, gf)	[13]	pRF4
pOX + nSlo2	[14]	Addgene plasmid #16202
pCAG *slo-2b(+)*	This paper	pIA097
pCAG *slo-2b(H159Y)*	This paper	pIA098
gcy-8p::slo-2b(H159Y)	This paper	pTAM005
gcy-8p::slo-2b(+)	This paper	pTAM011
snb-1p::slo-2b(H159Y)	This paper	pIA020
ttx-3p::slo-2b(H159Y)	This paper	pIA056
lin-11p::slo-2b(H159Y)	This paper	pIA057
glr-3p::slo-2b(H159Y)	This paper	pIA058
ceh-36p::slo-2b(H159Y)	This paper	pIA059
myo-3p::slo-2b(H159Y)	This paper	pIA060
cng-3 cDNA (partial)	Yuji Kohara	yk348a9
gcy-8p::cng-3(+)	This paper	pIA063
snb-1p::cng-3(+)	This paper	pIA064
ceh-36p::cng-3(+)	This paper	pIA065
pDESTttTi5605[R4-R3]	[15]	pCFJ150; Addgene plasmid #19329
Peft-3::Mos1 transposase	[8]	pCFJ601; Addgene plasmid #34874
Phsp::peel-1	[8]	pMA122; Addgene plasmid #34873
myo-2p::mCherry	[15]	pCFJ90; Addgene plasmid #19327
myo-3p::mCherry	[15]	pCFJ104; Addgene plasmid #19328
rab-3p::mCherry	[15]	pGH8; Addgene plasmid #19359
ttTi5605 gcy-8p::slo-1a(+)	This paper	pIA109
ttTi5605 gcy-8p::slo-1b(+)	This paper	pIA110
gcy-8p::slo-2b(R422H)	This paper	pTAM013
gcy-8p::slo-2b(Y732H)	This paper	pTAM014
gcy-8p::slo-2b(L219F)	This paper	pTAM015
gcy-8p::slo-2b(R376Q)	This paper	pTAM016
gcy-8p::slo-2b(I696M)	This paper	pTAM017
gcy-8p::slo-2b(R866C)	This paper	pTAM018
gcy-8p::slo-2b(F870I)	This paper	pTAM019
gcy-8p::mCherry::slo-2b(+)	This paper	pIA084
gcy-8p::mCherry::slo-2b(H159Y)	This paper	pIA085
gcy-8p::GFP::cng-3(+)	This paper	pIA094

Supplementary Methods.

RT-PCR.

First, mRNA was extracted from *C. elegans* using RNAiso PLUS reagent (Takara Bio, Kusatsu, Japan) and then it was subjected to reverse transcription by ReverTra Ace® qPCR RT Master Mix with gDNA Remover (Toyobo, Osaka, Japan). Then, cDNA was subjected to real-time PCR with THUNDERBIRD® SYBR qPCR Mix (Toyobo) and the CFX96 Real-Time PCR Detection System (Bio-Rad, Hercules, CA, USA). The sequences of primers used for qPCR are listed in Supplemental Table 1. The quantities of housekeeping genes such as *act-1, gpd-1,* and *lmn-1*, which encode actin, GAPDH, and lamine, respectively, were used as internal controls.

Supplementary References.

1. Hite, R. K. *et al.* Cryo-electron microscopy structure of the Slo2.2 Na+-activated K+ channel. *Nature* **527,** 198–203 (2015).
2. Wasserman, S. M., Beverly, M., Bell, H. W. & Sengupta, P. Regulation of response properties and operating range of the AFD thermosensory neurons by cGMP signaling. *Curr. Biol.* **21,** 353–362 (2011).
3. Wang, D., O'Halloran, D. & Goodman, M. B. GCY-8, PDE-2, and NCS-1 are critical elements of the cGMP-dependent thermotransduction cascade in the AFD neurons responsible for C. elegans thermotaxis. *J. Gen. Physiol.* **142,** 437–49 (2013).
4. Yu, Y. V. *et al.* CaMKI - Dependent Regulation of Sensory Gene Expression Mediates Experience - Dependent Plasticity in the Operating Range of a Thermosensory Neuron. *Neuron* **84,** 919–926 (2014).
5. Cho, S. W., Choi, K. Y. & Park, C. S. A new putative cyclic nucleotide-gated channel gene, cng-3, is critical for thermotolerance in Caenorhabditis elegans. *Biochem. Biophys. Res. Commun.* **325,** 525–531 (2004).
6. Davies, A. G. *et al.* A Central Role of the BK Potassium Channel in Behavioral Responses to Ethanol in C. elegans. *Cell* **115,** 655–666 (2003).
7. Wei, A. *et al.* Efficient isolation of targeted Caenorhabditis elegans deletion strains using highly thermostable restriction endonucleases and PCR. *Nucleic Acids Res.* **30,** e110 (2002).
8. Frøkjær-Jensen, C., Davis, M. W., Ailion, M. & Jorgensen, E. M. Improved Mos1-mediated transgenesis in C. elegans. *Nat Methods* **9,** 117–118 (2012).
9. C. elegans Deletion Mutant Consortium. large-scale screening for targeted knockouts in the Caenorhabditis elegans genome. *G3 (Bethesda).* **2,** 1415–25 (2012).
10. Kobayashi, K. *et al.* Single-Cell Memory Regulates a Neural Circuit for Sensory Behavior. *Cell Rep.* **14,** 11–21 (2016).
11. Friedland, A. E. *et al.* Heritable genome editing in C. elegans via a CRISPR-Cas9 system. *Nat. Methods* **10,** 741–3 (2013).
12. Kim, H. *et al.* A Co-CRISPR strategy for efficient genome editing in Caenorhabditis elegans. *Genetics* **197,** 1069–1080 (2014).
13. Mello, C. C., Kramer, J. M., Stinchcomb, D. & Ambros, V. Efficient gene transfer in C. elegans: extrachomosomal maintenance and integration of transforming sequences. *EMBO J.* **10,** 3959–3970 (1991).
14. Yuan, A. *et al.* SLO-2, a K+ channel with an unusual Cl- dependence. *Nat. Neurosci.* **3,** 771–779 (2000).
15. Frøkjær-Jensen, C. *et al.* Single copy insertion of transgenes in C. elegans. *Nat. Genet.* **40,** 1375–1383 (2008).
